# End-group-functionalized poly(*N*,*N*-diethylacrylamide) via free-radical chain transfer polymerization: Influence of sulfur oxidation and cyclodextrin on self-organization and cloud points in water

**DOI:** 10.3762/bjoc.10.61

**Published:** 2014-03-19

**Authors:** Sebastian Reinelt, Daniel Steinke, Helmut Ritter

**Affiliations:** 1 Heinrich-Heine-University of Düsseldorf, Institute of Organic Chemistry and Macromolecular Chemistry, Department of Preparative Polymer Chemistry, Universitätsstraße 1, 40225 Düsseldorf, Germany

**Keywords:** chain-transfer polymerization, cyclodextrins, end-group functionalization, host–guest interaction, lower critical solution temperature (LCST), poly(*N*,*N*-diethylacrylamide)

## Abstract

In this work we report the synthesis of thermo-, oxidation- and cyclodextrin- (CD) responsive end-group-functionalized polymers, based on *N*,*N*-diethylacrylamide (DEAAm). In a classical free-radical chain transfer polymerization, using thiol-functionalized 4-alkylphenols, namely 3-(4-(1,1-dimethylethan-1-yl)phenoxy)propane-1-thiol and 3-(4-(2,4,4-trimethylpentan-2-yl)phenoxy)propane-1-thiol, poly(*N*,*N*-diethylacrylamide) (PDEAAm) with well-defined hydrophobic end-groups is obtained. These end-group-functionalized polymers show different cloud point values, depending on the degree of polymerization and the presence of randomly methylated β-cyclodextrin (RAMEB-CD). Additionally, the influence of the oxidation of the incorporated thioether linkages on the cloud point is investigated. The resulting hydrophilic sulfoxides show higher cloud point values for the lower critical solution temperature (LCST). A high degree of functionalization is supported by ^1^H NMR-, SEC-, FTIR- and MALDI–TOF measurements.

## Introduction

Supramolecular chemistry was first defined by J. M. Lehn in the 1970`s as “chemistry of the intermolecular bond” [1–2]. However, its origin goes back to Fisher`s “lock and key” model and also to Watson and Cricks description of the role of H-bonds in DNA double helical structures. Both examples represent the importance of non-covalent interactions in living systems [3–4]. Since then, the field of self-assembly through molecular recognition has attracted much attention also in the design of smart materials. In this context, cyclodextrins (CD) are of interest as ring shaped host molecules, e.g., for the design of stimuli-responsive hydrogels [5] or of optical sensors [6]. Certain stimuli-responsive materials are characterized by the presence of thioethers in the main chain. Tirelli et al. investigated the oxidation-responsive behavior of thioethers for biomedical applications [7–10]. The stimulus of these mostly poly(propylene sulfide) containing copolymers is based on the oxidation of thioethers to more hydrophilic sulfoxides or sulfones [11–12]. The specific sulfoxidation of a polymer bound end-group, which is in the focus of our present work, has not yet been investigated.

Polymeric materials exhibiting sensitivity to temperature are widely investigated [13]. Within this group of materials, thermosensitive water-soluble polymers, possessing a lower critical solution temperature (LCST), have attracted much attention in several studies within the last decades [13–17].

The nature of the end-group of a short chain polymer may have a certain impact on the temperature dependence solubility in aqueous media [18–24]. There are two prevalent approaches to introduce well-defined end-groups in the polymer backbone: (a) direct introduction by the use of suitable initiators respectively chain-transfer agents [23–25] or (b) indirect by polymer analogous modification of existing end-groups. For the post-modification highly efficient reactions are needed to ensure a high degree of functionalization. For this reason, often “click reactions” [26] such as esterifications [27], azide–alkyne [22], thiol–ene [28], thiol–isocyanate [29] and others are used. Thereby most studies have in common that synthesis of the polymer with thermo-responsive properties is preferably accomplished by either living anionic polymerization, or controlled radical polymerization [18,21–22,24,29–34]. Some publications make use of free-radical chain transfer polymerization and subsequent polymer post-modification [25,27,35–36].

The scope of our investigation was the preparation of multiple-stimuli-responsive PDEAAm polymers possessing hydrophobic end-groups suitable for host–guest interactions with β-cyclodextrin derivatives. Since 4-alkylphenyl moieties are good guests for ß-cyclodextrin [37–38], we were encouraged to use 4-*tert*-butylphenol as well as 4-*tert*-octylphenol and modify them with mercapto groups. By doing so, well-defined PDEAAm end-group labeled polymers can be obtained by using classical free-radical chain transfer polymerization techniques. These polymers contain oxidation-sensitive thioether linkages. Up to now, to the best of our knowledge, the simultaneous influence of oxidation and cyclodextrin-sensitive end-groups on the solution properties of poly(*N*,*N*-diethylacrylamide) has not been investigated.

## Results and Discussion

**Scheme 1:**
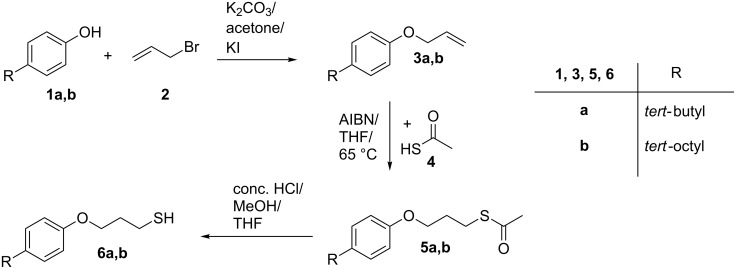
Synthetic route for the synthesis of thiol functionalized 4-alkylphenols.

**Synthesis of thiol functionalized 4-alkylphenols.** As depicted in Scheme 1, the synthesis of the thiol-functionalized phenol derivatives was accomplished in a three step synthesis. Etherification of the phenolic hydroxy groups of **1a** and **1b** with allyl bromide (**2**) and subsequent radical addition of ethanethioic *S*-acid (**4**) yielded the corresponding thioesters *S*-(3-(4-(1,1-dimethylethan-1-yl)phenoxy)propyl) ethanthioate (**5a**) and *S*-(3-(4-(2,4,4-trimethylpentan-2-yl)phenoxy)propyl) ethanthioate (**5b**). The thioester functions were hydrolyzed to obtain the thiols 3-(4-(1,1-dimethylethan-1-yl)phenoxy)propane-1-thiol (**6a**) and 3-(4-(2,4,4-trimethylpentan-2-yl)phenoxy)propane-1-thiol (**6b**) in good yields after purification. The successful synthesis was furthermore confirmed by ^1^H NMR, ^13^C NMR and FTIR spectroscopy as well as mass spectrometry (see Supporting Information File 1, Figures S1 to S4 for the ^1^H and ^13^C NMR data).

**Scheme 2:**
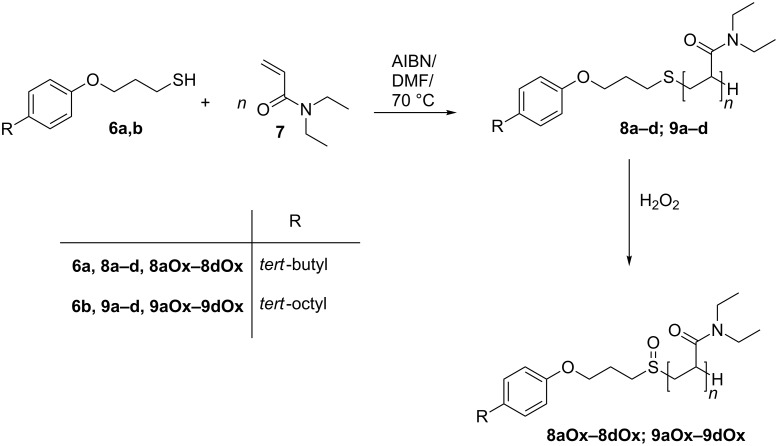
Synthetic route for the chain transfer polymerization of *N*,*N*-diethylacrylamide (**7**) with CTA **6a** and **6b** and subsequent oxidation of the resulting polymers to the corresponding polymers containing sulfoxides.

**Synthesis of the end-group functionalized polymers.** The thiol functionalized 4-alkylphenols (**6a** and **6b**) were used as chain transfer agents (CTA) for the free-radical polymerization of *N*,*N*-diethylacrylamide (DEAAm) (**7**) (see Scheme 2). First the chain-transfer constant of **6a** and **6b** for the polymerization of DEAAm in *N*,*N*-dimethylformamide (DMF) at 70 °C was calculated from experimental results by using the Mayo method [39]. Chain-transfer constants of C_Tr,6a_ = 0.84 and C_Tr,6b_ = 0.87 were found (see Supporting Information File 1, Figure S5), which are close to the ideal value of 1.0 where the concentration of the transfer agent relative to the monomer concentration remains constant [40].

For our investigation the molar ratio of [DEAAm] to [CTA] was varied from 20 to 1 up to 50 to 1 in order to investigate the influence of the chain length on the solubility in water. The molar ratio of [CTA] to [2,2’-azobis(2-methylpropionnitrile) (AIBN)] was kept thereby constant at a ratio of 20 to 1. The final polymers (**8a–d** and **9a–d**) were obtained as colorless solids after dialysis for 7 days. The analytical data of **8a–d** and **9a–d** are listed in Table 1.

**Table 1:** Number average molecular weights (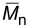), dispersity (D), glass transition temperatures (*T*_g_) and cloud points of the end-group-functionalized polymers (**8a–d** and **9a–d**).

Polymer	CTA	Ratio [DEAAm]/[CTA]	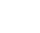 [kDa]^a^	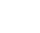 [kDa] (D)^b^	*T*_g_ [°C]	Cloud point [°C]^c^

**8a**	**6a**	20	2.5	3.3 (4.0)	49.1	15.1
**8b**	**6a**	30	4.8	5.6 (3.0)	62.3	21.2
**8c**	**6a**	40	6.1	5.7 (3.2)	65.9	24.3
**8d**	**6a**	50	7.8	7.2 (2.8)	75.3	25.8

**9a**	**6b**	20	2.9	3.4 (3.7)	50.4	21.3
**9b**	**6b**	30	4.4	5.4 (3.1)	63.9	25.2
**9c**	**6b**	40	5.9	5.2 (3.4)	73.2	27.6
**9d**	**6b**	50	7.2	6.9 (3.0)	75.4	28.7

^a^Determined by ^1^H NMR spectroscopy through end-group analysis; ^b^determined by size exclusion chromatography with DMF as eluent and a lower cut off of the column of 1.0 kDa; ^c^determined by turbidimetry measurements at a heating rate of 1 K/min. The concentration was 10 mg/mL in Millipore water. The cloud point values were derived from the heating curve.

**Figure 1:**
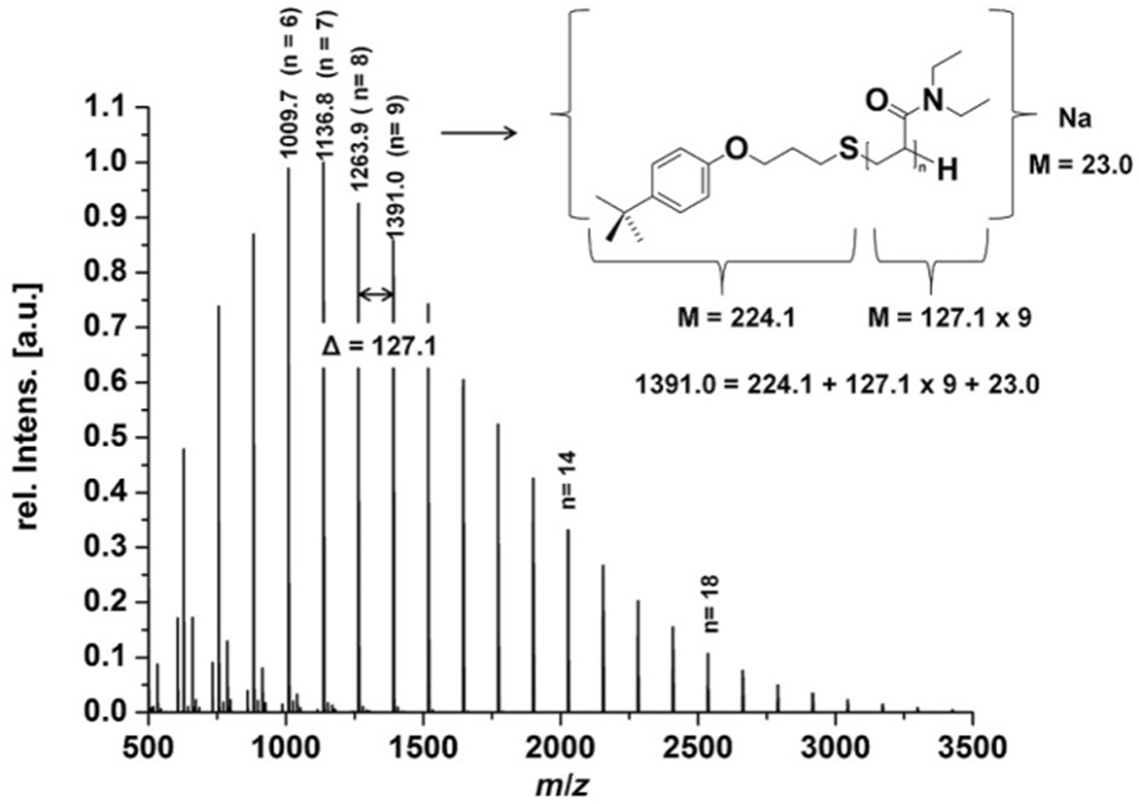
Section of the MALDI –TOF spectrum of polymer **8b**, indicating the high degree of end-group functionalization by the use of free-radical chain transfer polymerization.

**Figure 2:**
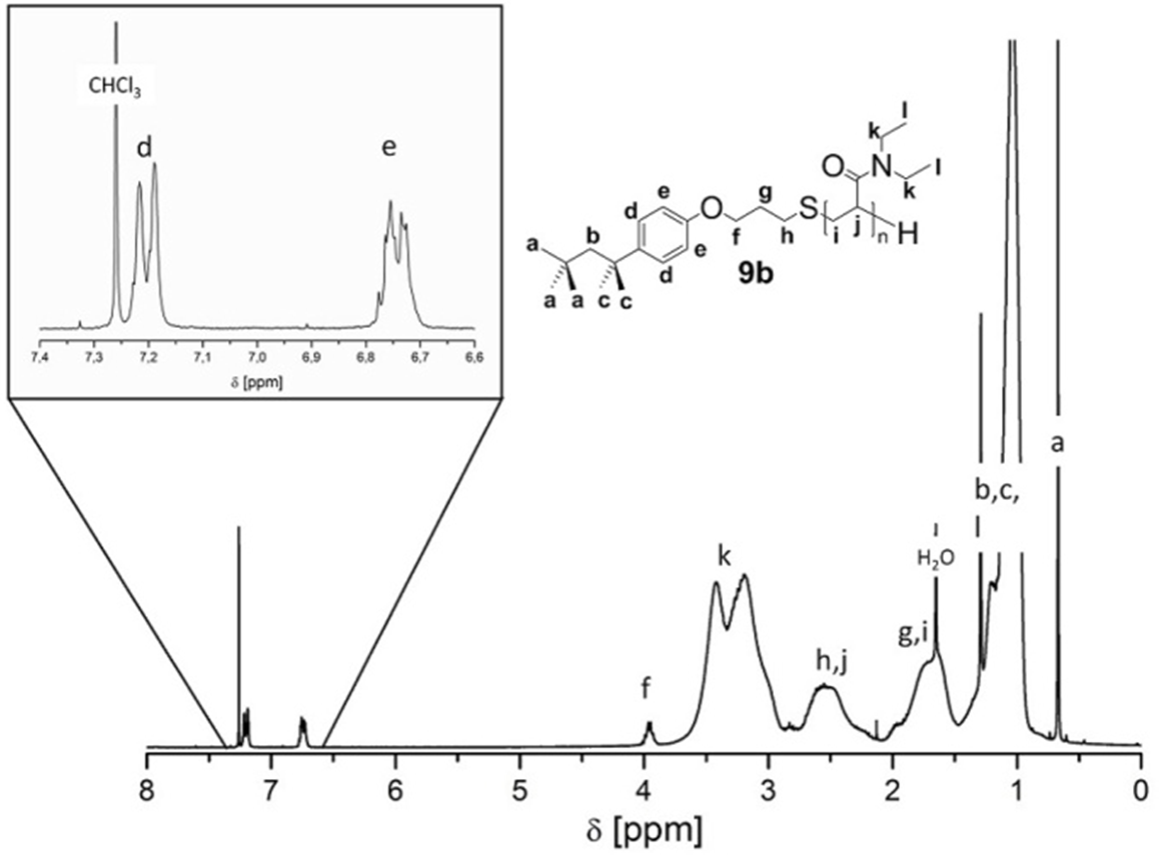
^1^H NMR spectrum of polymer **9b** in CDCl_3_ (300 MHz, rt).

Exemplarily, Figure 1 shows a section of the MALDI–TOF spectrum of polymer **8b** confirming a high degree of 4-*tert*-butylphenol end-group functionalization. Just single series of peaks with a peak separation of 127.1 which corresponds to the mass of DEAAm plus the proposed end-group (224.1) and sodium (23) can be found. Additionally, the 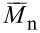 values determined from SEC data were in agreement with the values calculated by end-group analysis based on ^1^H NMR measurements (see Table 1). The NMR based 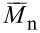 values were obtained by comparing the integral of aromatic signals at 6.7–6.8 ppm and 7.1–7.3 ppm with the signals of the backbone between 2.0–3.9 ppm and 0.7–2.0 ppm, respectively (see Figure 2 for polymer **9b** and Supporting Information File 1, Figure S6 for **8b**).

**Oxidation of the end-group-functionalized polymers.** The selective oxidation of the thioether groups to the corresponding sulfoxides was accomplished by oxidation with hydrogen peroxide in analogy to literature [11].

**Figure 3:**
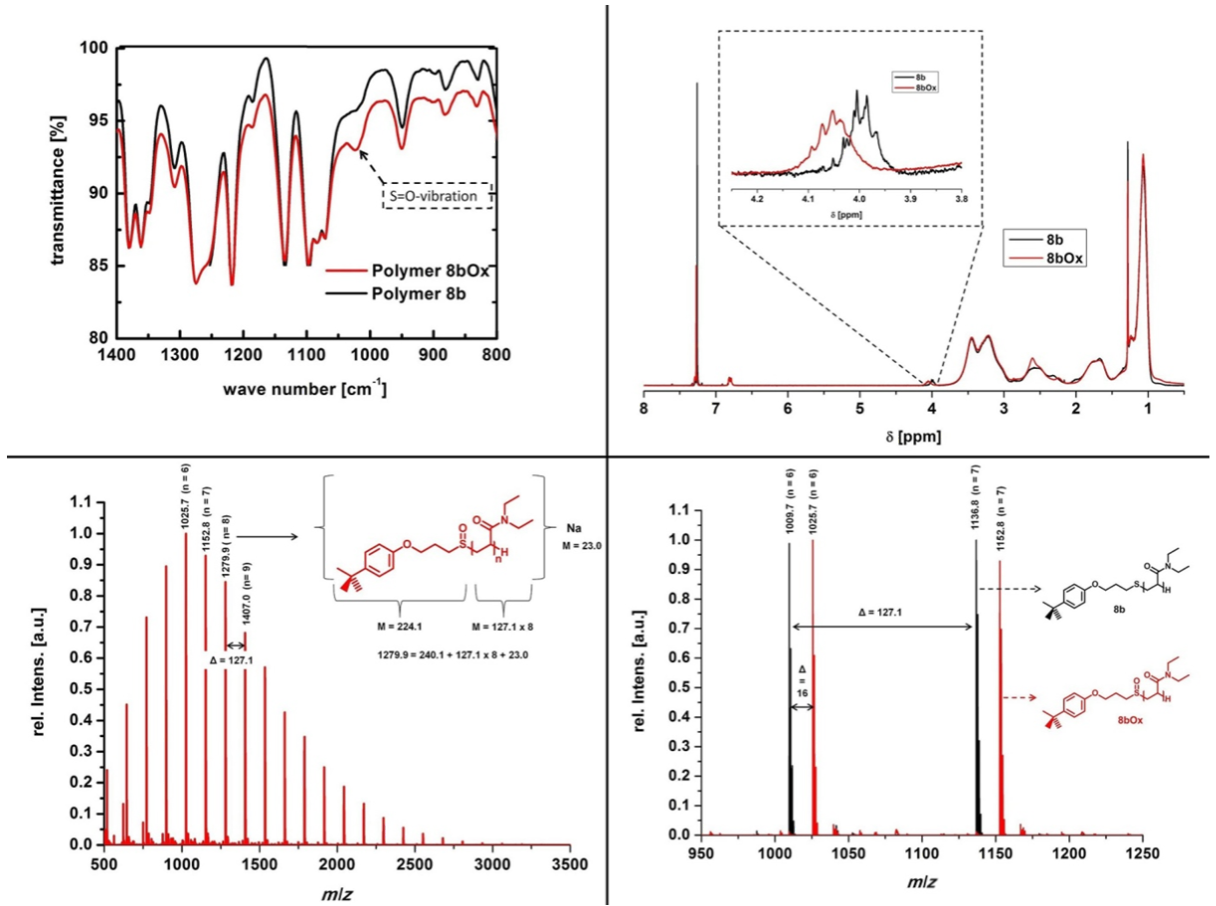
Top left: Section of the FTIR-spectrum of polymer **8b** (black line) in comparison to **8bOx** (red line); top right: ^1^H NMR spectrum of polymer **8b** (black line) and polymer **8bOx** (red line) in CDCl_3_ (300 MHz, rt); bottom left: Section of the MALDI–TOF spectrum of polymer **8bOx**, supporting the high efficiency of the oxidation of the thioether linkage to the corresponding sulfoxides; bottom right: Comparison of the MALDI–TOF spectrum of polymer **8b** (black line) with polymer **8bOx** (red line).

As expected, the MALDI–TOF mass spectrum for **8bOx** showed only one series of peaks, which was shifted by 16 Dalton in comparison to the origin series of peaks (see Figure 3). The FTIR spectrum showed a decrease of transmission at a wave length of 1024 cm^−1^ corresponding to the S=O stretching vibration of the sulfoxide [11] and no shift of the C=O vibration at 1625 cm^–1^. Additionally, the ^1^H NMR spectrum clearly indicated an upfield shift of the OCH_2_-group supporting also a successful oxidation. Thus the analytical data reveals no indication for further oxidation of the polymer chain or additional oxidized structures, e.g. of the methine groups in the main chain.

**Impact of the degree of polymerization and structure of the end-group on the cloud point values.** Aqueous solutions of poly(*N*,*N*-diethylacrylamide) (PDEAAm) exhibtit a coil to globule transition at the lower critical solution temperature (LCST) of approximately 33 °C in water for low molecular weight polymers as stated in previous studies [41–42]. In the present study, the cloud points of PDEAAm were measured on 1 wt % aqueous solution in water. A concentration of 1 wt % seemed to be a reasonable concentration since Idziak et al. [42] showed that a variation of the polymer concentration of PDEAAm (obtained in a free-radical polymerization with AIBN as initiator) in the range of 0.5 wt % up to 20 wt % does not considerably affect the LCST.

**Figure 4:**
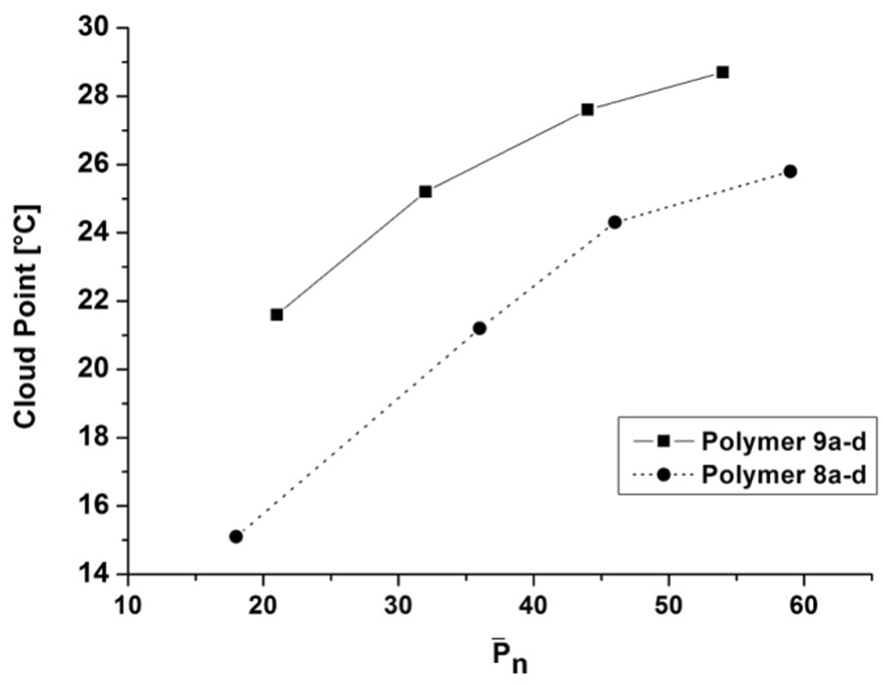
Dependency of the cloud point values on the degree of polymerization (calculated by end-group analysis based on ^1^H NMR measurements).

All obtained polymers (**8a–d, 9a–d**) were completely soluble in cold water below their LCST. Since the molecular weights of the presented polymers (**8a–d, 9a–d**) were relatively low, the hydrophobic end-groups shifted the cloud point significantly to lower temperatures in comparison to the cloud point of unmodified PDEAAm. Accordingly, the impact of the hydrophobic end-group on the cloud point of the polymer increased with decreasing molecular weight and thus showing an inverse dependency (Figure 4 and Table 2). This is in agreement with the findings of previous studies. As stated by Chung et al. the dehydration of the polymer chain during the phase transition is initiated at the chain ends, where the mobility is highest [36]. For poly(*N*-isopropylacrylamide) (PNIPAM) previous studies demonstrated a broadened phase transition for polydisperse and low molecular weight samples [18,32,43]. Although the polymers (**8a–d, 9a–d**) investigated in the present study were prepared via a free-radical instead of a living polymerization technique and had a relative low molecular weight, the optical transmission diagrams indicate a relatively sharp transition in a temperature range of 1 °C up to 2 °C as well as a good reversibility upon cooling (see Supporting Information File 1, Figures S7 and S8).

**Table 2:** Cloud points of the different polymers (**8a–d** and **9a–d**): Influence of RAMEB-CD and oxidation with H_2_O_2_.

Polymer	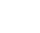 (*P*_n_) [kDa]^a^	Cloud Point [°C]^b^		Cloud Point after addition of RAMEB-CD [°C]^b^		

		Before oxidation	After oxidation^c^	1 equiv RAMEB-CD^d^	2 equiv RAMEB-CD^d^	4 equiv RAMEB-CD^d^

**8a**	2.5 (18)	15.1	21.0	27.9	34.2	34.2
**8b**	4.8 (36)	21.2	28.8	24.5	33.6	33.8
**8c**	6.1 (46)	24.3	30.0	26.2	33.4	33.4
**8d**	7.8 (59)	25.8	30.7	27.0	33.2	33.5

**9a**	2.9 (21)	21.3	24.3	–^e^	21.1	33.7
**9b**	4.4 (32)	25.2	27.4	–^e^	20.7	33.3
**9c**	5.9 (44)	27.6	28.2	19.6	22.4	33.2
**9d**	7.2 (54)	28.7	28.8	26.3	27.2	33.3

^a^Determined by ^1^H NMR spectroscopy through end-group analysis; ^b^determined by turbidimetry measurements at a heating rate of 1 K/min. The concentration was 10 mg/mL in Millipore water. The cloud point values were derived from the heating curve; ^c^oxidation was performed in aqueous hydrogen peroxide solution as stated in the experimental section; ^d^the stoichiometry was calculated on the basis of the data obtained from end-group analysis based on ^1^H NMR measurements; ^e^the polymer is insoluble in water, no optical clear solution down to 5 °C.

As it can be seen in Table 2 and Figure 4 the hydrophobic character caused by the chain-end was considerable for both types of end-groups. For instance, the cloud point of **8d** (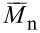 = 7.8 kDa) dropped from 25.8 °C to 15.1 °C for **8a** (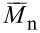 = 2.5 kDa) as the molecular weight decreased by 5.3 kDa. In contrast, with regard to cloud points of the 4-*tert*-octylphenol end-group functionalized polymers (**9a–d**) only a drop from 28.7 °C (**9d**, 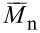 = 7.2 kDa) to 21.3 °C (**9a**, 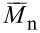 = 2.9 kDa) was found whereas the molecular weight decreased by 4.3 kDa. Our findings regarding the cloud point depression of thermoresponsive polymers containing hydrophobic end-groups with decreasing molecular weight [20,24,30,44] or the effect of an increasing hydrophobic environment in amphiphilic conetworks [45] are in accordance with previous studies. Theoretically a stronger remarkable effect on the cloud point of the polymers bearing a 4-*tert*-octylphenol end-group compared to the 4-*tert*-butylphenol end-group could be expected. Consequently hydrophobic interactions must play an important role for this effect. Regarding Figure 4 and Table 2 the hydrophobic interactions seem to be stronger for the 4-*tert*-octylphenol-modified polymers and thus leading to higher cloud points since aggregation leads to a suppression of the hydrophobicity of a polymer [25,35–36].

**Figure 5:**
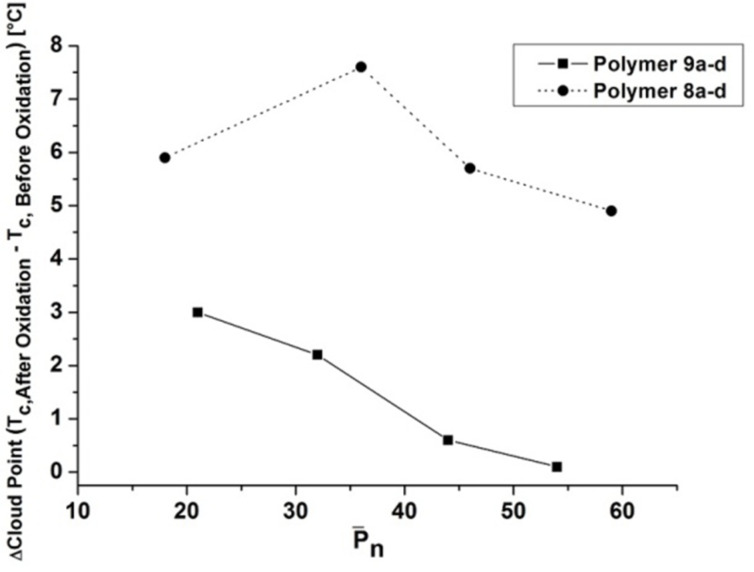
Shifts of the cloud points after the oxidation of the polymers (**8a–d, 9a–d**) to its corresponding sulfoxides (**8aOx–8dOx**, **9aOx–9dOx**) as a function of degree of polymerization.

**Figure 6:**
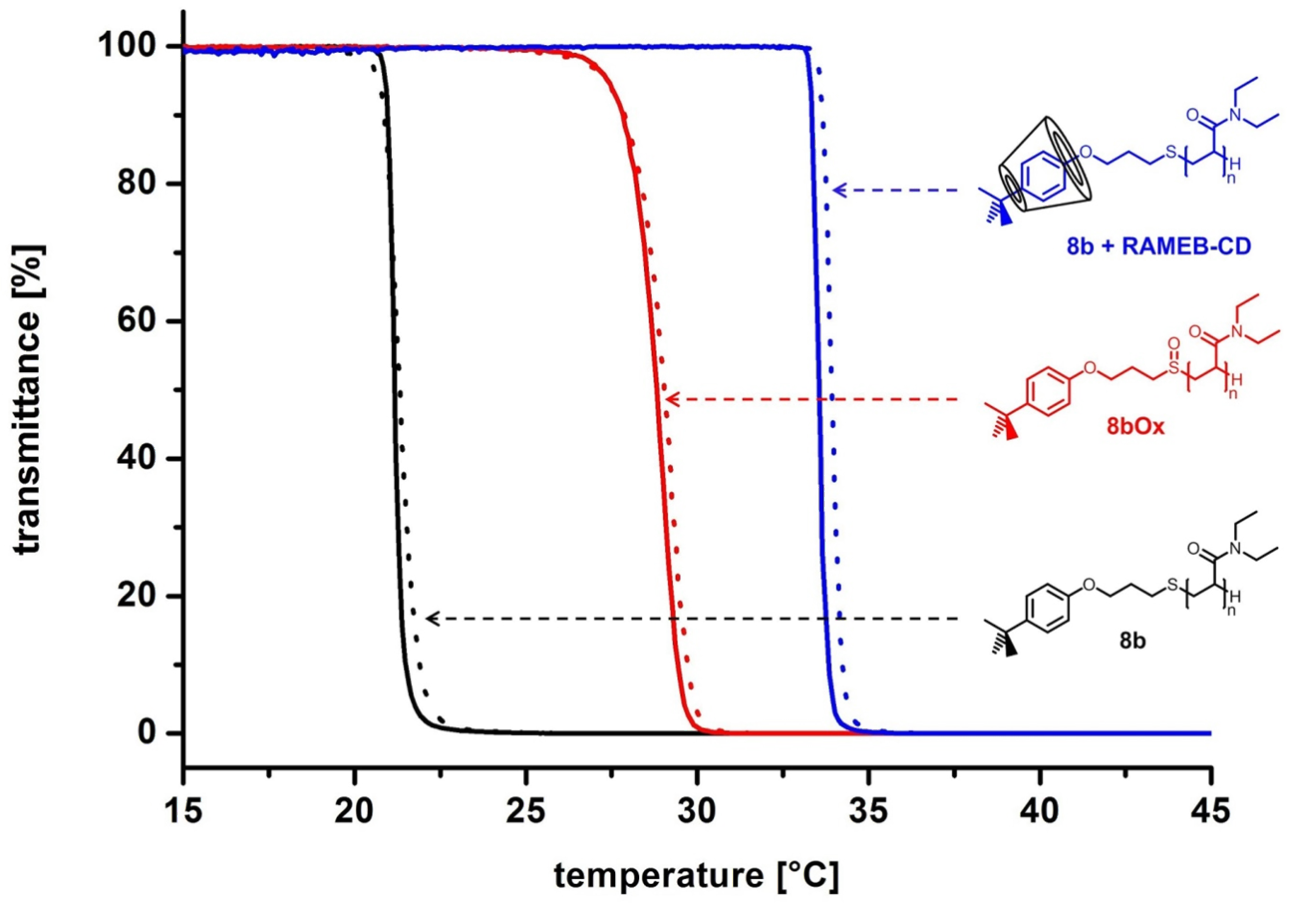
Turbidimetry measurements of polymer **8b** (straight black line – heating curve; dotted black line – cooling curve), **8bOx** (straight red line – heating curve; dotted red line – cooling curve) and the complex of **8b** and RAMEB-CD (2 equiv) (straight blue line – heating curve; dotted blue line – cooling curve) in aqueous solution at a concentration of 10 mg/mL.

**Impact of oxidation of the thioether-linkages on the solution properties.** Since the polymers bear thioether groups, the influence of their oxidation to sulfoxides on the cloud point was investigated. The more hydrophilic polar sulfoxide group in comparison to the thioether group should lead to an increase of solubility in water. Thus higher cloud point values for all polymers were found (see Table 2). The cloud point shifts are shown in Figure 5 for polymer **8** as well as **9**. The illustration indicates that the strength of response to oxidation of the aqueous solutions of polymers **8** and **9** is a function of the degree of polymerization. Thus the oxidation showed a more remarkable cloud point shift of the short chain polymers. Furthermore, it turned out, that the cloud points of the 4-*tert*-butylphenol end-group functionalized polymers (**8a–d**) were tunable to a larger extent via oxidation compared to the polymers bearing the 4-*tert*-octylphenol group. For instance polymer **8b** showed a threefold higher cloud point shift (7.6 °C) than the corresponding 4-*tert*-octylphenol-bearing polymer **9b** (2.2 °C). As an example, the cloud point curve of polymer **8b** before and after oxidation is illustrated in Figure 6.

**Figure 7:**
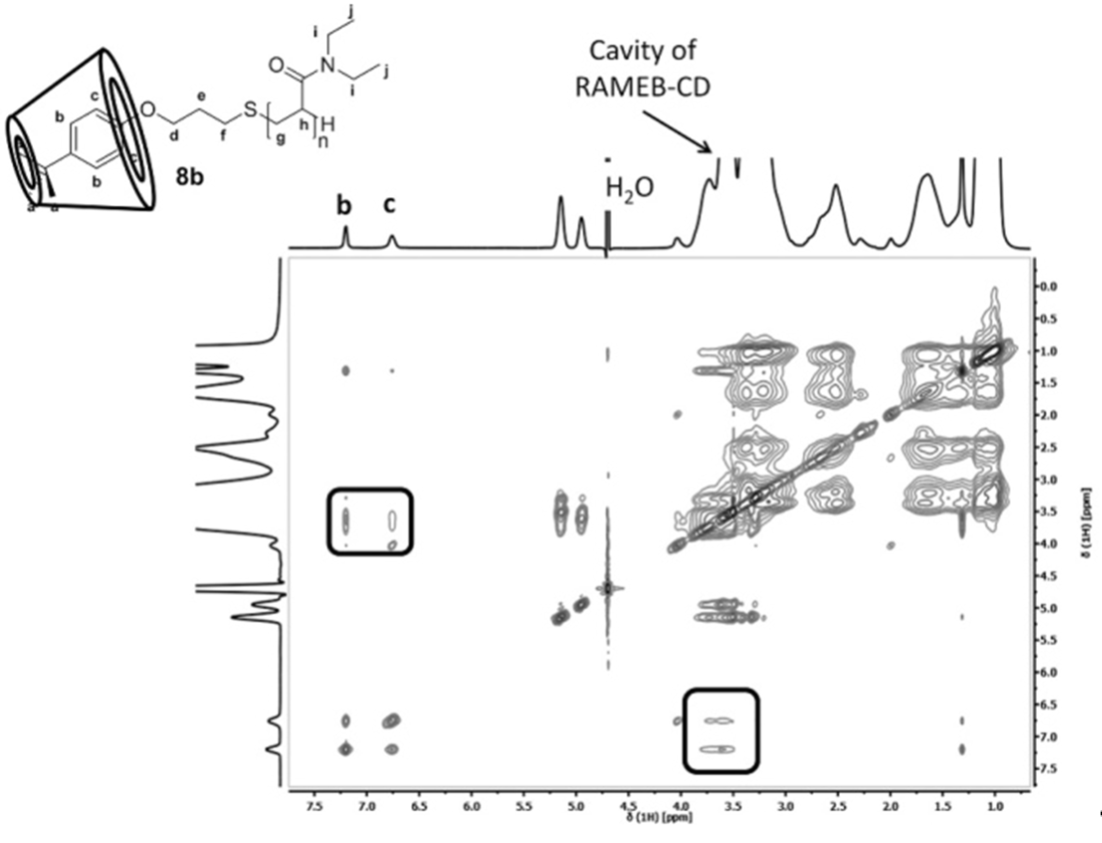
2D NMR NOESY spectrum of polymer **8b** with two equivalents RAMEB-CD in D_2_O (600 MHz, rt).

**Complexation of the polymer end-groups with randomly-methylated-β-cyclodextrin (RAMEB-CD) – Impact on the cloud points.** In addition the solution properties of polymers **8a–d** and **9a–d** as a function of temperature were also evaluated in the presence of different amounts of RAMEB-CD. In general, the 4-*tert*-butylphenyl as well as the 4-*tert*-octylphenyl end-group are able to build host-guest inclusion complexes with the RAMEB-CD cavity [38,46]. This interaction was verified by 2D NOESY NMR spectroscopy clearly showing correlation signals between the protons of the RAMEB-CD cavity and the aromatic protons as well as the aliphatic protons in case of the 4-*tert*-octyl end-group. Exemplarily, in Figure 7 the 2D NOESY spectrum of polymer **8b** in the presence of RAMEB-CD is shown.

The formation of host–guest complexes of the polymer bound end-group with the RAMEB-CD cavity should reduce the hydrophobic character of the end-group and thus increase the cloud points of the polymers to a value close to the value of pure PDEAAm as already shown by our group [24,47]. Taking a fourfold excess of RAMEB-CD in relation to the polymer end-group this assumption was fulfilled for all polymers (**8a–d** and **9a–d**). The values of the cloud point were increased after complexation up to the range of 33.2 °C and 34.2 °C, respectively (Table 2). However the addition of a twofold excess of RAMEB-CD to **8a–d** increased the cloud points also to values above 33 °C. Exemplarily, the shift of the cloud point curve of polymer **8b** after addition of two equivalents RAMEB-CD is illustrated in Figure 6. The addition of only an equimolar amount of RAMEB-CD caused an increase of the cloud points of **8a–d** up to values between 24.5 °C (**8b**) and 27.9 °C (**8a**) (Table 2). Due to the fact that the host–guest interactions are equilibria and the sterical accessibility of the polymer bound end-group is difficult, a slight excess of RAMEB-CD is necessary for the complete covering of each 4-*tert*-butylphenol end-group. These findings are in accordance with the literature [24,44].

The addition of equimolar amounts of RAMEB-CD to solutions of polymers **9a–d** containing 4-*tert*-octylphenol end-groups led to an unexpected decrease of the cloud point. This effect was most remarkable for the low molecular weight polymers **9a** and **9b**. For instance, a polymer **9a** was fully insoluble in water in the presence of one equivalent RAMEB-CD even at temperatures down to 5 °C. In case of polymer **9c** the cloud point dropped from 27.6 to 19.6 °C. Increasing the amount of RAMEB-CD led to an increase of the cloud points again.

**Figure 8:**
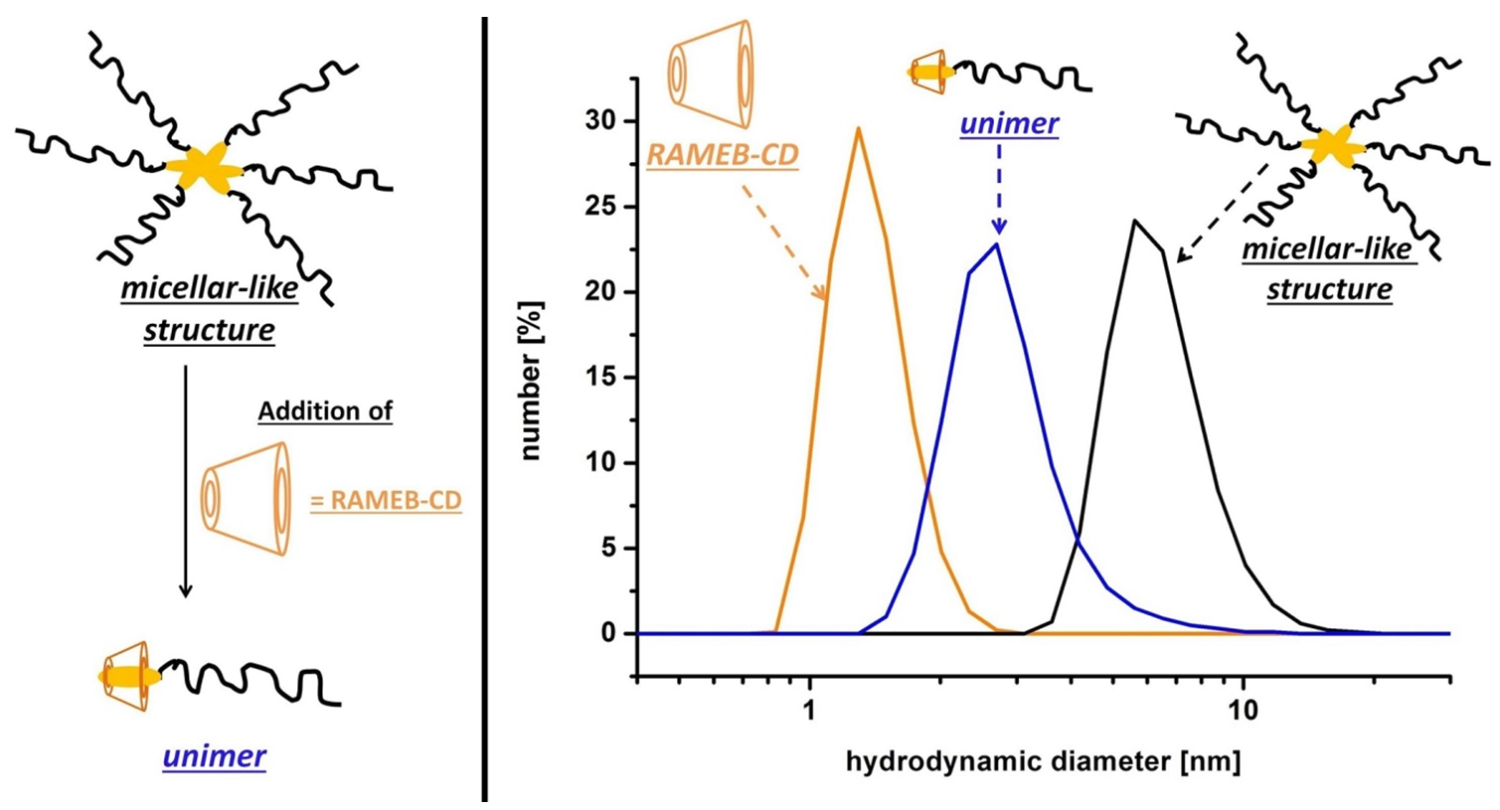
Left: Schematic illustration of the micellar-like structures and reversibility by addition of RAMEB-CD; Right: DLS measurements size distributions for RAMEB-CD (orange line), **9c** (black line) and **9c** plus 1 equiv RAMEB-CD (blue line).

**Dynamic light scattering measurements: Influence of RAMEB-CD on self-organization behavior.** Dynamic light scattering (DLS) measurements were conducted in order to understand the surprising phenomena that the cloud point of 4-*tert*-octylphenol end-group bearing polymers first decreased by adding small amounts of RAMEB-CD (one equivalent) to the aqueous polymer solution and subsequent increased again by rising the amount of RAMEB-CD up to four equivalents. The number averaged hydrodynamic diameters (*d_h_*) of the polymers **8a–d** and **9a–d** in aqueous solution (concentration: 10 mg/mL; temperature: 10 °C) were determined to 4.6 nm up to 6.8 nm. The addition of one equivalent RAMEB-CD to the aqueous polymer solutions of **8a–d** and **9c–d** led to a decrease of the number averaged hydrodynamic diameter (see Table 3). Exemplarily Figure 8 shows the hydrodynamic diameter of aqueous solutions of polymer **9c**, RAMEB-CD and polymer **9c** in the presence of one equivalent RAMEB-CD.

**Table 3:** Number averaged hydrodynamic diameters of the polymers and the supramolecular complexes with RAMEB-CD in water at 10 °C.

Polymer	Hydrodynamic diameter [nm]	

	10 mg/mL	10 mg/mL + 1 equiv RAMEB-CD

**8a**	6.3 ± 1.6	2.4 ± 0.8
**8b**	5.2 ± 1.5	3.6 ± 1.2
**8c**	5.4 ± 1.5	3.4 ± 1.2
**8d**	4.6 ± 1.5	3.4 ± 1.2
**9a**	6.8 ± 1.8	Insoluble^a^
**9b**	6.5 ± 1.7	Insoluble^a^
**9c**	6.5 ± 1.8	2.9 ± 1.0
**9d**	6.5 ± 1.7	3.1 ± 1.1

^a^At a temperature of 10 °C the solution was not optical clear, the sample is already aggregating.

These results indicated that the 4-*tert*-alkylphenol end-groups tend to aggregate strongly leading to the formation of intermolecular core-shell micellar-like structures as stated in Figure 8. However, it should be mentioned that especially after the addition of RAMEB-CD the intensity-weighted distributions showed also the formation of larger aggregates, but since the scattering intensity is dependent on the sixth power of the radius of the particle the percentage of these particles is exaggerated [48].

**Figure 9:**
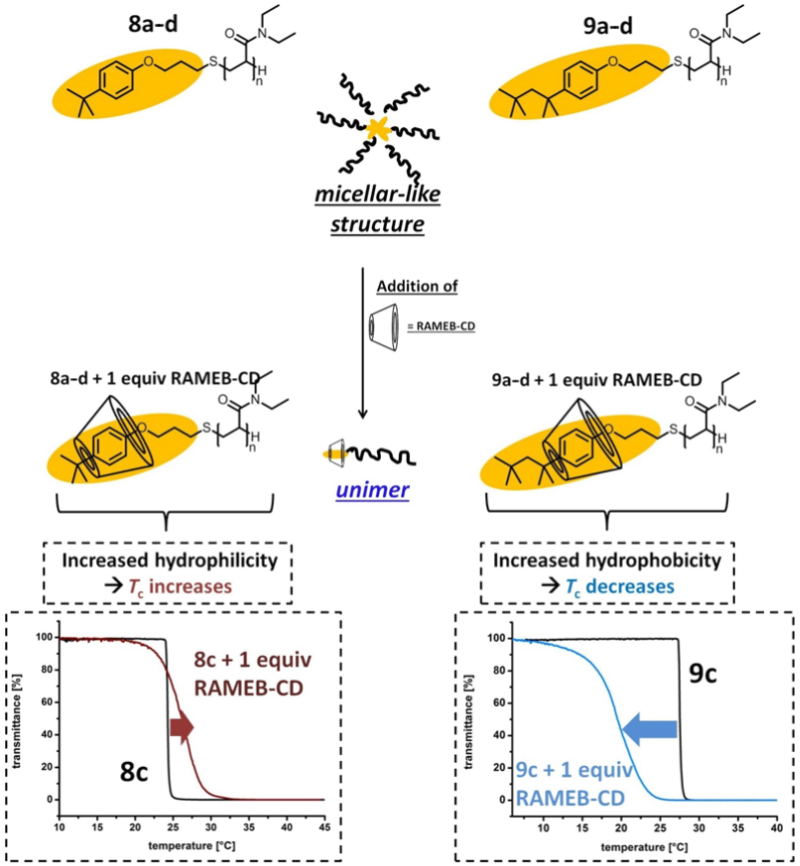
Schematic illustration of the micellar-like structures, its deformation upon addition of one equivalent RAMEB-CD and the influence on the cloud point values determined by turbitidy measurements on aqueous solutions (concentration: 10 mg/mL).

The formation of thermo-responsive micelles of end-group functionalized respectively block copolymers in aqueous solution have been investigated in previous studies [35,48–49]. Studies on hydrophobically PNIPAM have also demonstrated the formation of core-shell structures exhibiting a corona of PNIPAM chains [25,35–36]. The formation of micellar-like structures lead to an isolation of the hydrophobic end-groups from water and thus to a dramatically supression of the hydrophobicity of the polymer. Due to the interaction of the polymeric end-group with RAMEB-CD the formation of these core-shell structures is inhibited. The 4-*tert*-octylphenol end-group has a stronger hydrophobic character compared to the 4-*tert*-butylphenol group. Furthermore, it is well know that RAMEB-CD is able to build 2:1 complexes with 4-*tert*-octylphenol derivatives [46]. Thus it seems likely that one equivalent RAMEB-CD is not sufficient to depress the hydrophobic character of the 4-*tert*-octylphenol end-group completely, so that the micellar-like structures of **9a–d** in aqueous solution are more hydrophilic than the supramolecular complexes of **9a–d** with one equivalent RAMEB-CD. Consequently, as described above, a decrease of the cloud points of aqueous solutions of polymers **9a–d** were observed when adding only one equivalent RAMEB-CD. In case of the 4-*tert*-butylphenol modified polymers **8a–d** the supramolecular complexes are more hydrophilic than the micellar-like structures hence an increase of the cloud points were observed by addition of one equivalent RAMEB-CD. This phenomenon is illustrated in Figure 9.

## Conclusion

In summary, we have evidenced the synthesis and characterization of ω-(4-alyklphenyl)-functionalized PDEAAm via free-radical chain transfer polymerization in *N*,*N*-dimethylformamide as solvent. By changing the feed ratio of [CTA] to [DEAAm] the solution properties were tunable. A linear decrease of the cloud point with a decreasing degree of polymerization was observed. Aqueous polymer solutions showed multiple-stimuli-responsive behavior.

The cloud points of the aqueous polymer solutions were tunable by two different stimuli. The incorporated thioether linkages were addressable by oxidation. The corresponding more hydrophilic sulfoxides showed an increase of the cloud point values up to almost 8 °C depending on the end-group and the molecular weight of the polymer. Thus we were able to show for the first time the influence of sulfur oxidation of polymer bound end-groups on the solution properties in water. Simultaneously the cloud points were tunable by the addition of RAMEB-CD. The cloud points of all polymers (**8a–d, 9a–d**) could shift to a value of 33–34 °C in case of RAMEB-CD in excess.

## Supporting Information

A full experimental section can be found in the Supporting Information. Description of the materials, characterization methods and syntheses of the obtained compounds; spectroscopic data (^1^H, ^13^C and 2D NMR); curves of the turbidity measurements, Mayo-Plot for the determination of the chain transfer constant.

File 1 Title Experimental part.
