# Supplementary material for: End-group-functionalized poly(N,N-diethylacrylamide) via free-radical chain transfer polymerization: Influence of sulfur oxidation and cyclodextrin on self-organization and cloud points in water
Source: Beilstein J Org Chem. 2014 Mar 19;10:680–91. doi: 10.3762/bjoc.10.61 (PMC3999829; doi:10.3762/bjoc.10.61)
Supplement: File 1 — Title Experimental part. [file Beilstein_J_Org_Chem-10-680-s001.pdf]

Supporting Information

for

End-group-functionalized poly(*N*,*N*-diethylacrylamide) via free-radical chain transfer polymerization: Influence of sulfur oxidation and cyclodextrin on self-organization and cloud points in water

Sebastian Reinelt, Daniel Steinke and Helmut Ritter*

Address: Heinrich-Heine-University of Düsseldorf, Institute of Organic Chemistry and Macromolecular Chemistry, Department of Preparative Polymer Chemistry, Universitätsstraße 1, 40225 Düsseldorf, Germany.

Email: Helmut Ritter - H.Ritter@uni-duesseldorf.de

*corresponding author

Experimental part

Materials and methods

Materials. 2,2'-azobis(2-methylpropionitrile) (Acros Organics, 98%), 4-*tert*-butylphenol (Sigma Aldrich, 99%), 4-*tert*-octylphenol (Sigma Aldrich, 97%), *N*,*N*-diethylacrylamide (TCI Europe, 98%), acetone (Sigma Aldrich, p.a.), allyl bromide (Acros Organics, 99%), ethanethioic S-acid (Acros Organics, 98%), hydrochloric acid 37% (VWR, normapur), hydrogen peroxide 30% (AppliChem, p.a.), methanol (Fisher Chemicals, p.a.), sodium hydroxide (AppliChem, p.a.), potassium carbonate (Sigma Aldrich, p.a.), tetrahydrofuran (VWR, normapur) were used as received. RAMEB-CD was obtained from Wacker Chemie GmbH, Burghausen, Germany and was used after being dried overnight with a vacuum oil pump over P4O10. Chloroform-*d* (99.8 atom % D) and deuterium oxide (99.9 atom % D) was obtained from Deutero GmbH, Germany. As not stated different, all solvents and other chemicals used were of analytical grade and were used as received without any further purification.

Characterization methods. ^{1}H NMR and ^{13}C NMR measurements were performed on a Bruker Avance 300 or 600 spectrometer operating at 300 and 600 MHz for ^{1}H and at 75 and 150 MHz for ^{13}C-experiments at room temperature. The signals of the uncompleted deuterated solvents were used as internal standard.

For elementary analyses the elemental analyzer vario MICRO Cube (Elementar Analysensysteme GmbH) was used.

Infrared spectra were recorded on a Nicolet 6700 FT-IR spectrometer equipped with a diamond single bounce ATR accessory. The measurements were performed in the range of 4000–300 cm^{-1} at room temperature.

Differential scanning calorimetry (DSC) analysis was conducted with a differential scanning calorimeter Mettler Toledo DSC 822 with a sample robot TSO801RO. The experiments were performed in temperature range from 20 °C to 200 °C at a scan rate of 15 K/min. The T_g values are reported as the average of three measurement upon heating using the midpoint method.

Gas chromatography coupled with electron impact ionization mass spectrometry (GC/MS (EI)) was performed on a GC–MS-System comprising a triple quadrupole ion trap mass spectrometer Finnigan Trace DSQ and a Finnigan Trace GC Ultra. The instrument was calibrated in the *m*/*z* range of 4000 Da. Matrix-assisted laser desorption-ionization time-of-flight mass spectrometry (MALDI–TOF–MS) was performed on a Bruker Ultraflex time of flight mass spectrometer. Ions formed with a pulsed nitrogen laser (25 Hz, 337 nm) were accelerated to 25 kV, the molecular masses being recorded in the linear mode. Dithranol was used as a matrix and sodium trifluoroacetate (NaTFA) as ionization agent.

Size exclusion chromatography was performed using a ViscotekGPCmax VE2001 system. The system has a column set compromising one MZ-Gel-SDplus, 100 Å pore size and 10 µm particle size, 50 × 8.0 mm [Length × ID] pre-column and two MZ-Gel-SDplus linear, 10 µm particle size , 300 × 8.0 mm [Length × ID] columns. The columns were constantly heated to a temperature of 60 °C. *N*,*N*-Dimethylformamide (0.05 M LiBr) was used as eluent at a flow rate of 1 mL/min. For detection a Viscotek VE 3500 RI detector was used. The system was calibrated with polystyrene standards with a molecular range from 1,280 g/mol to 1,373,300 g/mol.

Turbidity experiments were performed on a Tepper cloud point photometer TP1. The degree of turbidity was measured by a power-regulated semiconductor laser with a wavelength of 670 nm and a silicon photodiode. The experiments were performed in a temperature range between 10

and 45 °C and a heating rate of 1 °C min^{-1} by using Hellma Suprasil precision cells 110 Q-S. All critical solution temperatures were detected by determination of the temperature where the transparency of the solution was 50 % of the initial value upon heating. As not stated different – all measurements were done with a concentration of 10 mg/mL in millipore water. All solutions were freshly prepared one day before use.

Dynamic light scattering (DLS) was recorded in backscattering mode on a Malvern Zetasizer Nano ZS ZEN 3600 at a temperature of 20 °C with a laser wavelength of 633 nm and a detection angle of 173°. Measured solutions contained 10 mg/mL of the polymer in millipore water and were performed in a glass cuvette QS with a layer thickness of 1 cm. The non-negative-least-squares algorithm in the general-purpose mode was used for interpretation. Each experiment was performed at least 10 times to obtain statistical information and all hydrodynamic diameters (D_h) are the averages of the number weighted distributions.

Synthesis of the chain transfer agents (6a and 6b)

Synthesis of 1-allyloxy-4-(1,1-dimethylethan-1-yl)benzene (3a) [1]

In a 250 mL three-neck round-bottom flask 4-*tert*-butylphenol (**1a**, 15.04 g, 100 mmol), potassium carbonate (9.69 g, 70 mmol) and potassium iodide (0.5 g, 0.3 mmol) were dissolved in 120 mL acetone. After heating the white suspension to reflux for 60 minutes under an nitrogen atmosphere, allyl bromide (**2**, 15.72 g, 130 mmol) dissolved in 30 mL acetone was added through a dropping funnel. The mixture was stirred for 40 hours under reflux. Then 100 mL of deionized water was added and extracted with diethyl ether (3 × 80 mL). The combined organic extracts were washed with 1 N sodium hydroxide solution (3 × 50 mL) and brine (50 mL), dried over magnesium sulfate, filtered and concentrated under reduced pressure. The product **3a** (16.03 g, 84 mmol, 84% [Lit: 94%]) was obtained as pale yellow liquid.

^{1}H NMR (300 MHz, $CDCl_3$, δ [*ppm*]): 7.36 – 7.28 (m, 2H, Ar-***H***), 6.92 – 6.85 (m, 2H, Ar-***H***), 6.09 (ddt, *J* = 17.3 Hz / 10.6 Hz / 5.3 Hz, 1H, $-OCH_2C\boldsymbol{H}=CH_2$), 5.48 – 5.39 (m, 1H, $-OCH_2CH=C\boldsymbol{H}_2$), 5.33 – 5.27 (m, 1H, $-OCH_2CH=C\boldsymbol{H}_2$), 4.57 – 4.53 (m, 1H, $-OC\boldsymbol{H}_2CH=CH_2$), 1.32 (s, 9H, $-C(C\boldsymbol{H}_3)_3$).

Synthesis of 1-allyloxy-4-(2,4,4-trimethylpentan-2-yl)benzene (3b) [2]

In a 250 mL three-neck round-bottom flask 4-*tert*-octylphenol (**1b**, 15.44 g, 75 mmol), potassium carbonate (9.27 g, 68 mmol) and potassium iodide (0.5 g, 0.3 mmol) were dissolved in 120 mL acetone. After heating the white suspension to reflux for 60 minutes under an atmosphere of nitrogen, allyl bromide (**2**, 14.41 g, 119 mmol) dissolved in 30 mL acetone was added through a dropping funnel. The mixture was stirred for 40 hours under reflux. Then 100

mL of deionized water was added and extracted with diethyl ether (3 × 80 mL). The combined organic extracts were washed with saturated sodium hydrogen carbonate solution (3 × 50 mL) and brine (50 mL), dried over magnesium sulfate, filtered and concentrated under reduced pressure. The product **3b** (16.91 g, 69 mmol, 92% [Lit: 78%]) was obtained as pale yellow liquid.

^{1}H NMR (300 MHz, $CDCl_3$, δ [*ppm*]): 7.35 – 7.22 (m, 2H, Ar-***H***), 6.92 – 6.80 (m, 2H, Ar-***H***), 6.09 (ddt, *J* = 17.3 Hz / 10.5 Hz / 6.1 Hz, 1H, -$OCH_2C\boldsymbol{H}=CH_2$), 5.50 – 5.36 (m, 1H, -$OCH_2CH=C\boldsymbol{H_2}$), 5.35 – 5.23 (m, 1H, -$OCH_2CH=C\boldsymbol{H_2}$), 4.58 – 4.49 (m, 1H, -$OC\boldsymbol{H_2}CH=CH_2$), 1.73 (s, 2H, -$C(CH_3)_2C\boldsymbol{H_2}C(CH_3)_3$), 1.37 (s, 6H, -$C(C\boldsymbol{H_3})_2CH_2C(CH_3)_3$), 0.74 (s, 9H, -$C(CH_3)_2CH_2C(C\boldsymbol{H_3})_3$); GC/MS (EI) *m*/*z* (%): 246 (8) [M^+], 176 (18), 175 (100) [$M^+ - C_5H_{11}$].

Synthesis of *S*-(3-(4-(1,1-dimethylethan-1-yl)phenoxy)propyl) ethanthioate (5a)

In a 250 mL three-neck round-bottom flask 1-allyloxy-4-(1,1-dimethylethan-1-yl)benzene **3a** (15.22 g, 80 mmol), ethanethioic *S*-acid (**4**, 12.25 g, 161 mmol) and 2,2`-azobis(2-methylpropionitrile) (AIBN) (0.66 g, 4 mmol) were dissolved in 100 mL tetrahydrofuran. The reaction mixture was purged with nitrogen for 30 minutes at ambient temperature and then stirred at 60 °C for 16 h under a nitrogen atmosphere. After cooling the reaction mixture in an ice bath to 0 °C, a 1 N sodium carbonate solution (80 mL) was added and extracted with dichloromethane (3 × 80 mL). The combined organic extracts were washed with 1 N sodium hydroxide solution (3 × 50 mL) and saturated brine (1 × 50 mL), dried over magnesium sulfate, filtered and concentrated under reduced pressure. The residual oil was purified via column chromatography on silica gel with ethylacetate/*n*-hexane 1:9 as eluent (R_f = 0.45). The product **5a** (17.8 g, 67 mmol, 84%) was obtained as slightly yellow liquid.

^{1}H NMR (300 MHz, $CDCl_3$, δ [*ppm*]): 7.36 – 7.29 (m, 2H, Ar-***H***), 6.89 – 6.83 (m, 2H, Ar-***H***), 4.10 (t, *J* = 6.1 Hz, 2H, -OC$\boldsymbol{H_2}$-), 3.09 (t, *J* = 6.1 Hz, 2H, -$CH_2C\boldsymbol{H_2}S$-), 2.36 (s, 3H, -S(C=O)C$\boldsymbol{H_3}$), 2.09 (tt, *J* = 7.1 Hz / 6.1 Hz, 2H, -C$\boldsymbol{H_2}$-), 1,33 (s, 9H, -C(C$\boldsymbol{H_3}$)$_3$); ^{13}C NMR (75 MHz, $CDCl_3$, δ [*ppm*]): 195.71 (***C***=O), 156.58 (Ar-***C1***), 143.48 (Ar-***C4***), 126.27 (Ar-***C2,C6***), 114.02 (Ar-***C3,C5***), 66.16 (-O***C***H_2-), 34.12 (-***C***$(CH_3)_3$), 31.61 (-C(***C***$H_3)_3$), 30.69 (-(C=O)***C***H_3), 29.44 (-***C***H_2S-), 26.01 (-***C***H_2-); FT-IR: *ν* = 3034 (w, *ν*(CH(Ar))), 2959 (m, $\nu_{as}(CH_3)$), 2905 (w, $\nu_{as}(CH_2)$), 2868 (w, $\nu_s(CH_3)$), 1690 (s, *ν*(C=O)), 1609 (m), 1581 (w), 1512 (s, *ν*(C=C)), 1470 (m, $\delta_s(CH_2)$), 1391 (w), 1362 (m, $\delta_s(CH_3)$), 1294 (m, $\delta(C(CH_3)_3)$, 1242 (s, *ν*(C-O)), 1183 (s, *ν*(C-O)), 1133 (s), 1109 (m), 1040 (m), 955 (m, *ν*(C-S)), 828 (s, δ(Ar-H)), 622 (s) cm^{-1}; GC/MS (EI) *m*/*z* (%): 266 (9) [M^+], 135 (24), 117 (100) [$C_5H_9OS^+$], 75 (10), 43 (17).

Synthesis of S-(3-(4-(2,4,4-trimethylpentan-2-yl)phenoxy)propyl) ethanthioate (5b)

In a 250 mL three-neck round-bottom flask 1-allyloxy-4-(2,4,4-trimethylpentan-2-yl)benzene **3b** (14.76 g, 60 mmol), ethanethioic *S*-acid (**4**, 9.20 g, 121 mmol) and 2,2`-azobis(2-methylpropionitrile) (AIBN) (0.49 g, 3 mmol) were dissolved in 100 mL tetrahydrofuran. The reaction mixture was purged with nitrogen for 30 minutes at ambient temperature and then stirred at 60 °C for 16 h under a nitrogen atmosphere. After cooling the reaction mixture in an ice bath to 0 °C, a 1 N sodium carbonate solution (80 mL) was added and extracted with dichloromethane (3 × 80 mL). The combined organic extracts were washed with 1 N sodium hydroxide solution (3 × 50 mL) and saturated brine (1 × 50 mL), dried over magnesium sulfate, filtered and concentrated under reduced pressure. The residual oil was purified via column chromatography on silica gel with ethylacetate/*n*-hexane 1:19 (R_f = 0.38) as eluent. The product **5b** (14.25 g, 44 mmol, 73%) was obtained as slightly yellow liquid.

^{1}H NMR (300 MHz, $CDCl_3$, δ [*ppm*]): 7.31– 7.25 (m, 2H, Ar-***H***), 6.85 – 6.81 (m, 2H, Ar-***H***), 4.02 (t, *J* = 6.1 Hz, 2H; -OC***H₂***-), 3.09 (t, *J* = 7.1 Hz, 2H, -C***H₂***S-), 2.36 (s, 3H, -S(C=O)C***H₃***), 2.08 (tt, *J* = 7.1 Hz / 6.1 Hz, 2H, -OCH₂C***H₂***-), 1.73 (s, 2H, -C(CH₃)₂C***H₂***C(CH₃)₃), 1.37 (s, 6H, -C(C***H₃***)₂CH₂C(CH₃)₃), 0.75 (s, 9H, -C(CH₃)₂CH₂C(C***H₃***)₃); ^{13}C NMR (75 MHz, $CDCl_3$, δ [*ppm*]): 195.85 (***C***=O), 156.51 (Ar-***C1***), 142.46 (Ar-***C4***), 127.16 (Ar-***C2,C6***), 113.78 (Ar-***C3,C5***), 66.21 (-O***C***H₂-), 57.11 (-C(CH₃)₂***C***H₂C(CH₃)₃), 38.07 (-***C***(CH₃)₂CH₂C(CH₃)₃), 32.44 (-C(CH₃)₂CH₂***C***(CH₃)₃), 31.90 (-C(CH₃)₂CH₂C(***C***H₃)₃), 31.80 (-C(***C***H₃)₂CH₂C(CH₃)₃), 30.76 (-(C=O)***C***H₃), 29.52 (-***C***H₂S-), 26.09 (-OCH₂***C***H₂-); FT-IR: *ν* = 3034 (w, *ν*(CH(Ar))), 2952 (m, $\nu_{as}(CH_3)$), 2901 (w, $\nu_{as}(CH_2)$), 2867 (w, $\nu_s(CH_3)$), 1692 (s, *ν*(C=O)), 1610 (m), 1579 (w), 1511 (s, *ν*(C=C)), 1471 (m, $\delta_s(CH_2)$), 1394 (w), 1385 (w), 1364 (m, $\delta_s(CH_3)$), 1295 (m, $\delta(C(CH_3)_3)$), 1237 (s, *ν*(C-O)), 1184 (s, *ν*(C-O)), 1132 (s), 1109 (m), 1041 (m), 955 (m, *ν*(C-S)), 827 (s, *δ*(Ar-H)), 623 (s) cm^{-1}; GC/MS (EI) *m*/*z* (%): 322 (4) [M^+], 251 (41) [$M^+ - C_5H_{11}$], 135 (17), 117 (100) [$C_5H_9OS^+$], 57 (19), 43 (19).

Synthesis of 3-(4-(1,1-dimetyhlethan-1-yl)phenoxy)propane-1-thiol (6a)

In a 250 mL three-neck round-bottom flask *S*-(3-(4-*tert*-butyl)phenoxy)propyl) ethanthioate (**5a**, 16.16 g, 61 mmol) and concentrated hydrochloric acid (6.5 g) were dissolved in 120 mL methanol and 40 mL tetrahydrofuran. The resulting solution was degassed by bubbling with nitrogen for 30 minutes, afterwards heated to 60 °C and stirred at 60 °C for 24 h under a nitrogen atmosphere. After cooling the reaction mixture deionized water (50 mL) was added and extracted with dichloromethane (3 × 100 mL). The combined organic extracts were washed with saturated sodium hydrogen carbonate solution (1 × 50 mL) and brine (3 × 40 mL), dried over magnesium

sulfate, filtered and concentrated under reduced pressure. Bulb-to-bulb distillation (130 °C, 10^{-2} mbar) yielded the product **6a** (10.6 g, 47 mmol, 77%) as colourless liquid.

^{1}H NMR (300 MHz, $CDCl_3$, δ [*ppm*]): 7.38– 7.31 (m, 2H, Ar-***H***), 6.92 – 6.85 (m, 2H, Ar-***H***), 4.10 (t, *J* = 5.9 Hz, 2H; -OC$\boldsymbol{H_2}$-), 2.76 (dt, *J* = 8.1 Hz / 7.0 Hz, 2H, -C$\boldsymbol{H_2}$SH), 2.10 (tt, *J* = 7.0 Hz / 5.9 Hz, 2H, -C$\boldsymbol{H_2}$-), 1.42 (t, *J* = 8.1 Hz, 1H, -CH_2S***H***), 1.37 (s, 9H, -C(C$\boldsymbol{H_3}$)$_3$); ^{13}C NMR (75 MHz, $CDCl_3$, δ [*ppm*]): 156.63 (Ar-***C1***), 143.51 (Ar-***C4***), 126.31 (Ar-***C2,C6***), 114.02 (Ar-***C3,C5***), 65.63 (-O***C***H_2-), 34.15 (-***C***(CH_3)$_3$), 33.54 (-***C***H_2-), 31.63 (-C(***C***H_3)$_3$), 21.40 (-***C***H_2SH); FT-IR: ν = 3039 (w, ν(CH(Ar))), 2960 (m, $\nu_{as}(CH_3)$), 2901 (w, $\nu_{as}(CH_2)$), 2867 (w, $\nu_s(CH_3)$), 2563 (w, ν(S-H)), 1609 (m), 1580 (w), 1512 (s, ν(C=C)), 1470 (m, $\delta_s(CH_2)$), 1391 (w), 1363 (m, $\delta_s(CH_3)$), 1294 (m, $\delta(C(CH_3)_3)$, 1241 (s, ν(C-O)), 1183 (s, ν(C-O)), 1117 (w), 1037 (m), 965 (m, ν(C-S)), 828 (s, δ(Ar-H)) cm^{-1}; GC/MS (EI) *m*/*z* (%): 224 (51) [M^+], 209 (60) [$M^+ - CH_3$], 35 (100), 107 (13); Anal. calc. for $C_{13}H_{20}OS$: C 69.59, H 8.98; found: C 69.02, H 8.90.

Synthesis of 3-(4-(2,4,4-trimethylpentan-2-yl)phenoxy)propane-1-thiol (6b)

In a 250 mL two-neck round-bottom flask *S*-(3-(4-(2,4,4-trimethylpentan-2-yl)phenoxy)propyl) ethanthioate (**5b**, 12.90 g, 40 mmol) and concentrated hydrochloric acid (4.3 g) were dissolved in 120 mL methanol and 40 mL tetrahydrofuran. The resulting solution was degassed by bubbling with nitrogen for 30 minutes and afterwards heated to 60 °C and stirred at 60 °C for 24 h under a nitrogen atmosphere. After cooling the reaction mixture deionized water (50 mL) was added and extracted with dichloromethane (3 × 100 mL). The combined organic extracts were washed with saturated sodium hydrogen carbonate solution (1 × 40 mL) and brine (2 × 40 mL), dried over magnesium sulfate, filtered and concentrated under reduced pressure. The residual oil was purified via column chromatography on silica gel with ethylacetate/*n*-

hexane 1:9 (R_f = 0.61) as eluent. The product **6b** (8.81 g, 31 mmol, 78%) was obtained as colourless liquid.

^{1}H NMR (300 MHz, $CDCl_3$, δ [*ppm*]): 7.31– 7.23 (m, 2H, Ar-***H***), 6.85 – 6.78 (m, 2H, Ar-***H***), 4.06 (t, *J* = 5.9 Hz, 2H; -OC$\boldsymbol{H_2}$-), 2.74 (dt, *J* = 8.1 Hz / 7.0 Hz, 2H, -C$\boldsymbol{H_2}$SH), 2.07 (tt, *J* = 7.0 Hz / 5.9 Hz, 2H, -OCH_2C$\boldsymbol{H_2}$-), 1.71 (s, 2H, -$C(CH_3)_2$C$\boldsymbol{H_2}$$C(CH_3)_3$), 1.40 (t, *J* = 7.0 Hz, 1H, -CH_2S***H***), 1.35 (s, 6H, -C(C$\boldsymbol{H_3}$)$_2$$CH_2C(CH_3)_3$), 0.72 (s, 9H, -$C(CH_3)_2CH_2$C(C$\boldsymbol{H_3}$)$_3$); ^{13}C NMR (75 MHz, $CDCl_3$, δ [*ppm*]): 156.57 (Ar-***C1***), 142.50 (Ar-***C4***), 127.20 (Ar-***C2,C6***), 113.78 (Ar-***C3,C5***), 65.68 (-O***C***H_2-), 57.12 (-$C(CH_3)_2$***C***$H_2C(CH_3)_3$), 38.09 (-***C***$(CH_3)_2CH_2C(CH_3)_3$), 33.65 (-OCH_2***C***H_2-), 32.47 (-$C(CH_3)_2CH_2$***C***$(CH_3)_3$), 31.92 (-$C(CH_3)_2CH_2C$(***C***H_3)$_3$), 31.82 (-C(***C***H_3)$_2CH_2C(CH_3)_3$), 21.49 (-***C***H_2SH); FT-IR: *ν* = 3039 (w, *ν*(CH(Ar))), 2951 (m, $\nu_{as}(CH_3)$), 2901 (w, $\nu_{as}(CH_2)$), 2867 (w, $\nu_s(CH_3)$), 2569 (w, *ν*(S-H)), 1610 (m), 1580 (w), 1510 (s, *ν*(C=C)), 1470 (m, $\delta_s(CH_2)$), 1385 (w), 1364 (m, $\delta_s(CH_3)$), 1295 (m, $\delta(C(CH_3)_3)$), 1241 (s, *ν*(C-O)), 1184 (s, *ν*(C-O)), 1038 (m), 966 (w, *ν*(C-S)), 827 (s, *δ*(Ar-H)) cm^{-1}; GC/MS (EI) *m*/*z* (%): 280 (8) [M^+], 209 (100) [$M^+ - C_5H_{11}$], 210 (14), 135 (74), 107 (12), 57 (15); Anal. calc. for $C_{13}H_{20}OS$: C 72.80, H 10.06; found: C 72.77, H 10.01.

Determination of the chain-transfer constant

The chain transfer constants were determined by using the Mayo-equation [3,4]:

$$1/DP_n = C_{Tr}\,[Thiol]/[Monomer] + 1/DP_{n,0} \quad (1)$$

The polymerization was carried out at constant concentration of 2,2'-azobis(2-methylpropionnitrile) (AIBN) and *N*,*N*-diethylacrylamide (DEAAm) in *N*,*N*-dimethylformamide (DMF) as solvent at 70 °C. Different concentrations of the thiol **6a** or **6b** were used. The reciprocal degree of polymerization (DP_n) – determined by SEC – was plotted against the ratio of the concentrations of **6a**/**6b** and DEAAm. The slope of the line afforded the chain transfer constant (C_{Tr}) according to equation 1.

General procedure for the chain-transfer polymerizations in solution

After dissolving DEAAm, the chain-transfer agent (**6a** or **6b**) and AIBN (see Table 1) in DMF (16.5 wt %) the resulting solution was degassed by bubbling with nitrogen for 30 minutes. Afterwards the solution was placed into a preheated oil bath at a temperature of 70 °C. After 16 hours at 70 °C the reaction mixture was cooled with liquid nitrogen and quenched by exposure to air. *N*,*N*-dimethylformamide was removed under reduced pressure. The crude product was dissolved in 20 mL of destilled water and afterwards dialysed with a Spectra/Por 6 dialysis membrane from Spectrum Laboratories (MWCO = 1000 Da) for 7 days at ambient temperature against water. The resulting solution was freeze dried to yield a white solid.

Table S1. Synthesis of the end-group functionalized polymers (**8a–d** and **9a–d**)

Resulting Polymer	Thiol	Amount of thiol [mg]	Amount of DEAAM [g]	Amount of AIBN [mg]	Amount of DMF [mL]	Yield [g] (%)
**8a**	**6a**	341	3.867	12.5	20.6	3.50 (83%)
**8b**	**6a**	247	4.196	9.0	22.4	4.11 (92 %)
**8c**	**6a**	203	4.607	7.4	24.5	3.57 (74 %)
**8d**	**6a**	152	4.317	5.6	23.0	3.23 (72 %)
**9a**	**6b**	369	3.347	10.8	17.8	3.35 (90 %)
**9b**	**6b**	345	4.698	10.1	25.0	4.08 (80 %)
**9c**	**6b**	246	4.460	7.2	23.8	3.34 (71 %)
**9d**	**6b**	156	3.541	4.6	18.9	3.06 (82 %)

General procedure for the oxidation of the end-group-functionalized polymers

500 mg of each polymer **8a–d and 9a–d** was placed in a 50 mL round-bottom flask and dissolved in 15 mL Millipore water. To the stirred solution the 30-fold excess of a 30% hydrogen peroxide solution corresponding to the end-group analysis based on the ^{1}H NMR measurements was added. The closed flasks were stirred for 7 days at ambient temperature. Afterwards the solvent was evaporated under reduced pressure and the polymers were dried in a vacuum oven at a temperature of 70 °C for 10 hours. The oxidized polymers **8aOx–8dOx** and **9aOx–9dOx** were obtained as a quantitative yield of white solids.

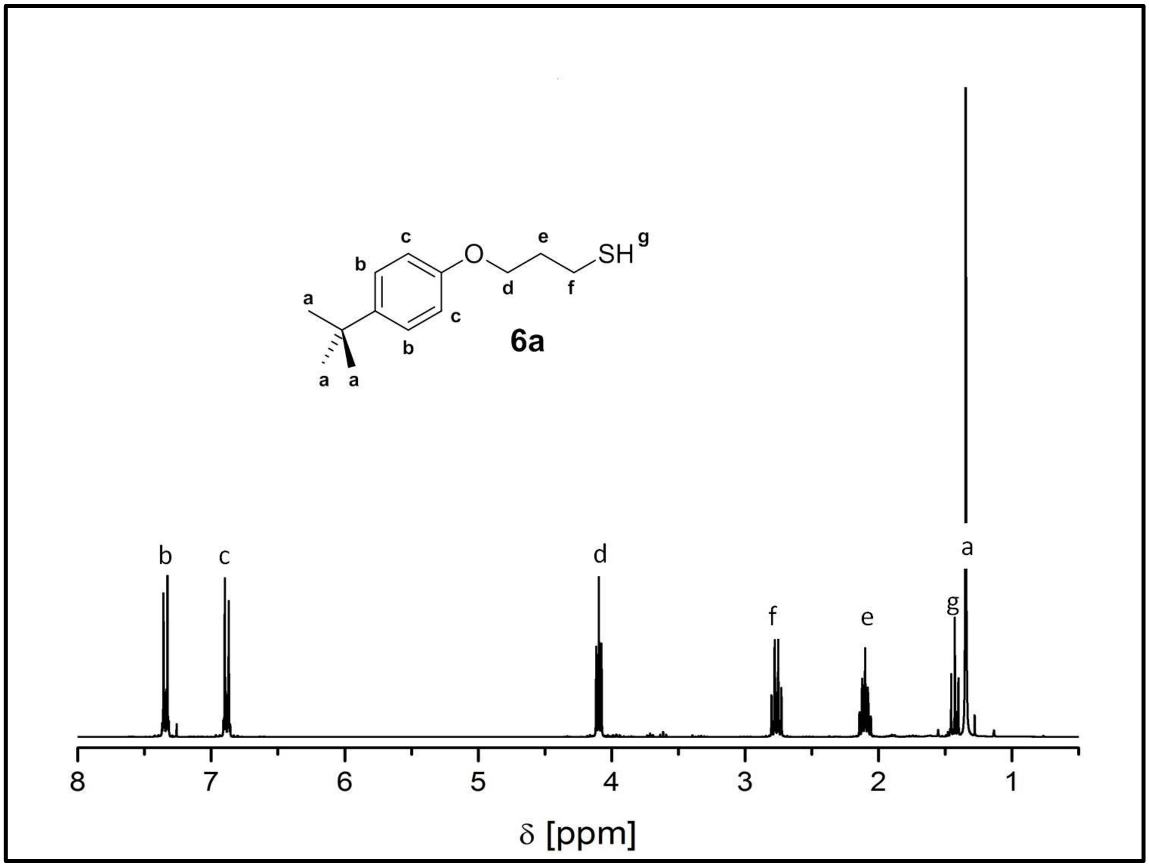

Figure S1: ^{1}H NMR spectrum of 3-(4-(1,1-dimetyhlethan-1-yl)phenoxy)propane-1-thiol (**6a**) in $CDCl_3$ (300 MHz, rt).

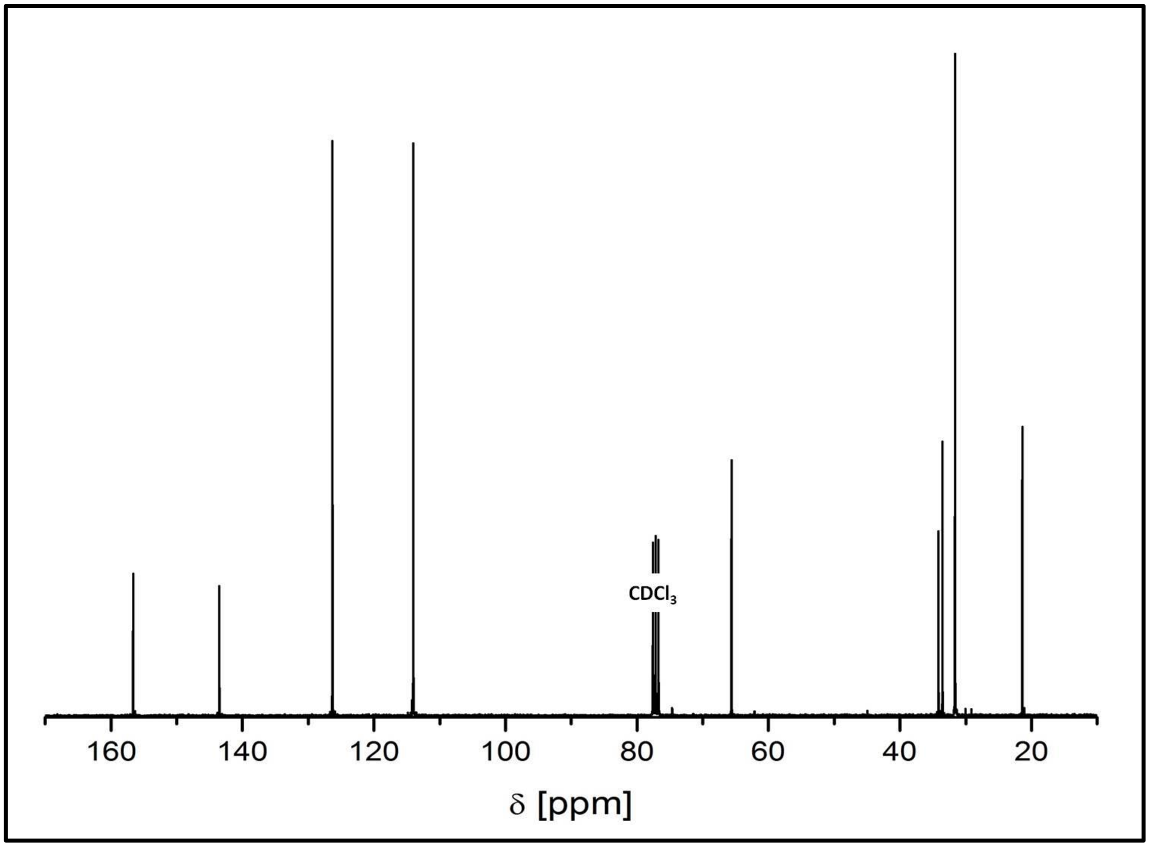

Figure S2: ^{13}C NMR spectrum of 3-(4-(1,1-dimetyhlethan-1-yl)phenoxy)propane-1-thiol (**6a**) in $CDCl_3$ (75 MHz, rt).

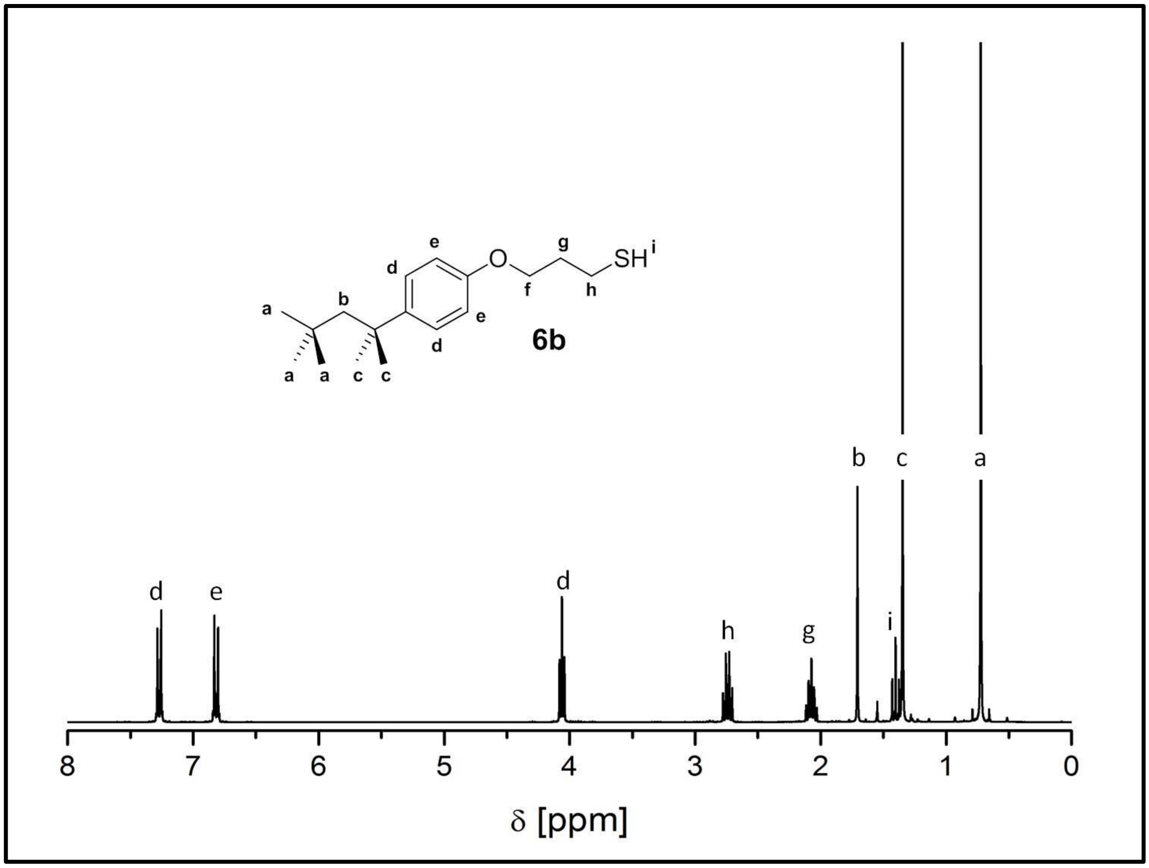

Figure S3: ^{1}H NMR spectrum of 3-(4-(2,4,4-trimethylpentan-2-yl)phenoxy)propyl)propane-1-thiol (**6b**) in $CDCl_3$ (300 MHz, rt).

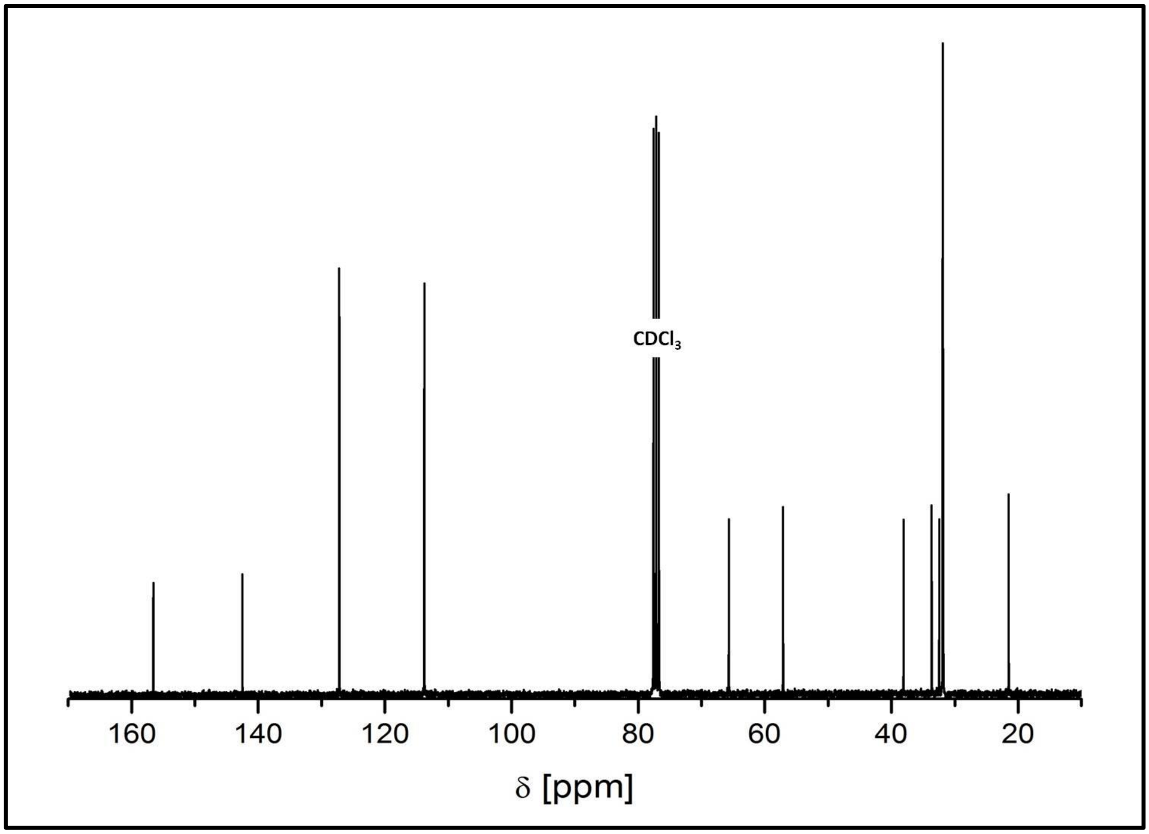

Figure S4: ^{13}C NMR spectrum of 3-(4-(2,4,4-trimethylpentan-2-yl)phenoxy)propyl)propane-1-thiol (**6b**) in $CDCl_3$ (75 MHz, rt).

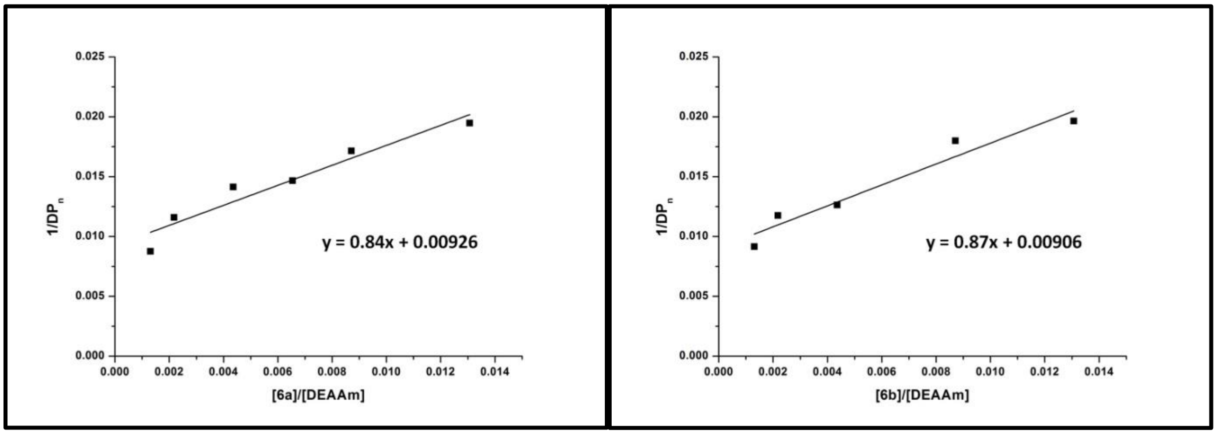

Figure S5: Determination of the chain transfer constant according to the Mayo method.

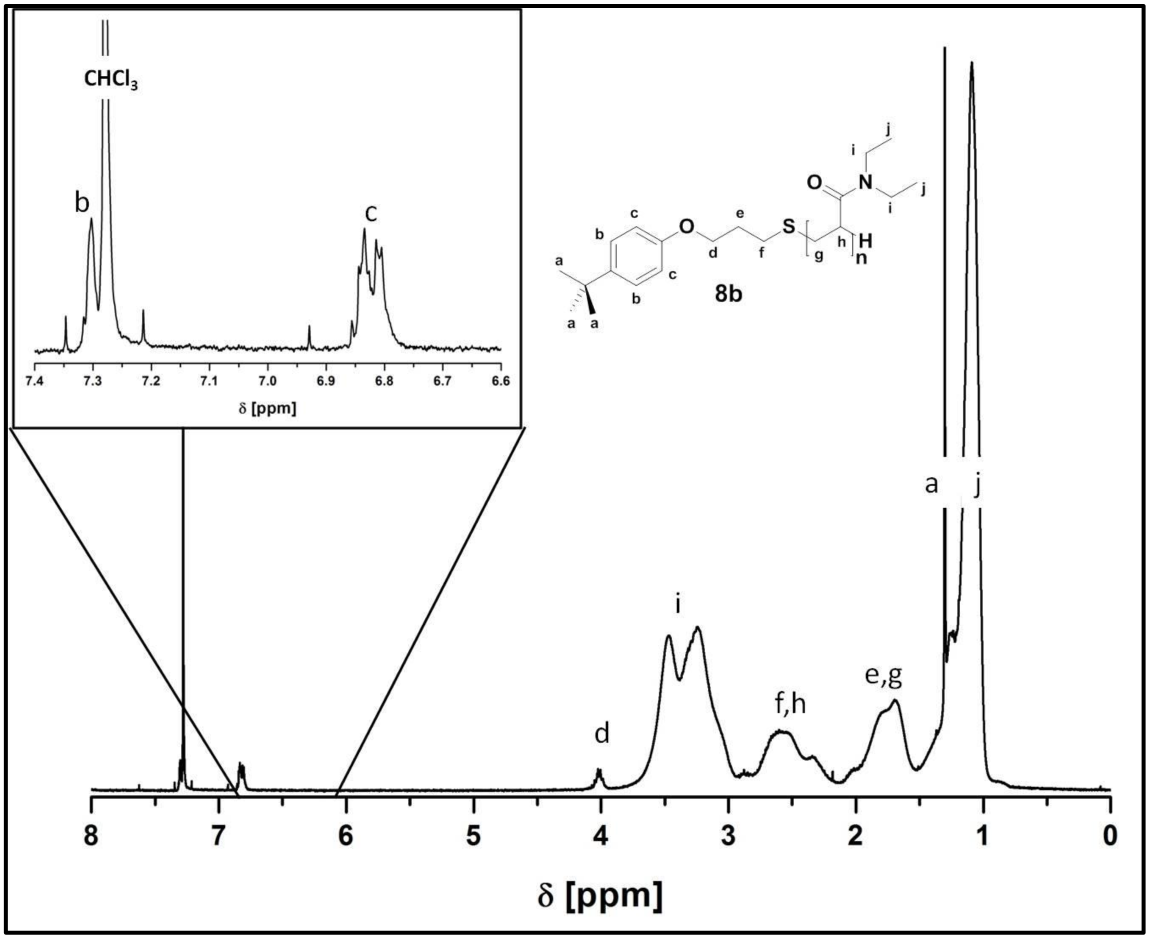

Figure S6: ^{1}H NMR spectrum of polymer **8b** in $CDCl_3$ (300 MHz, rt).

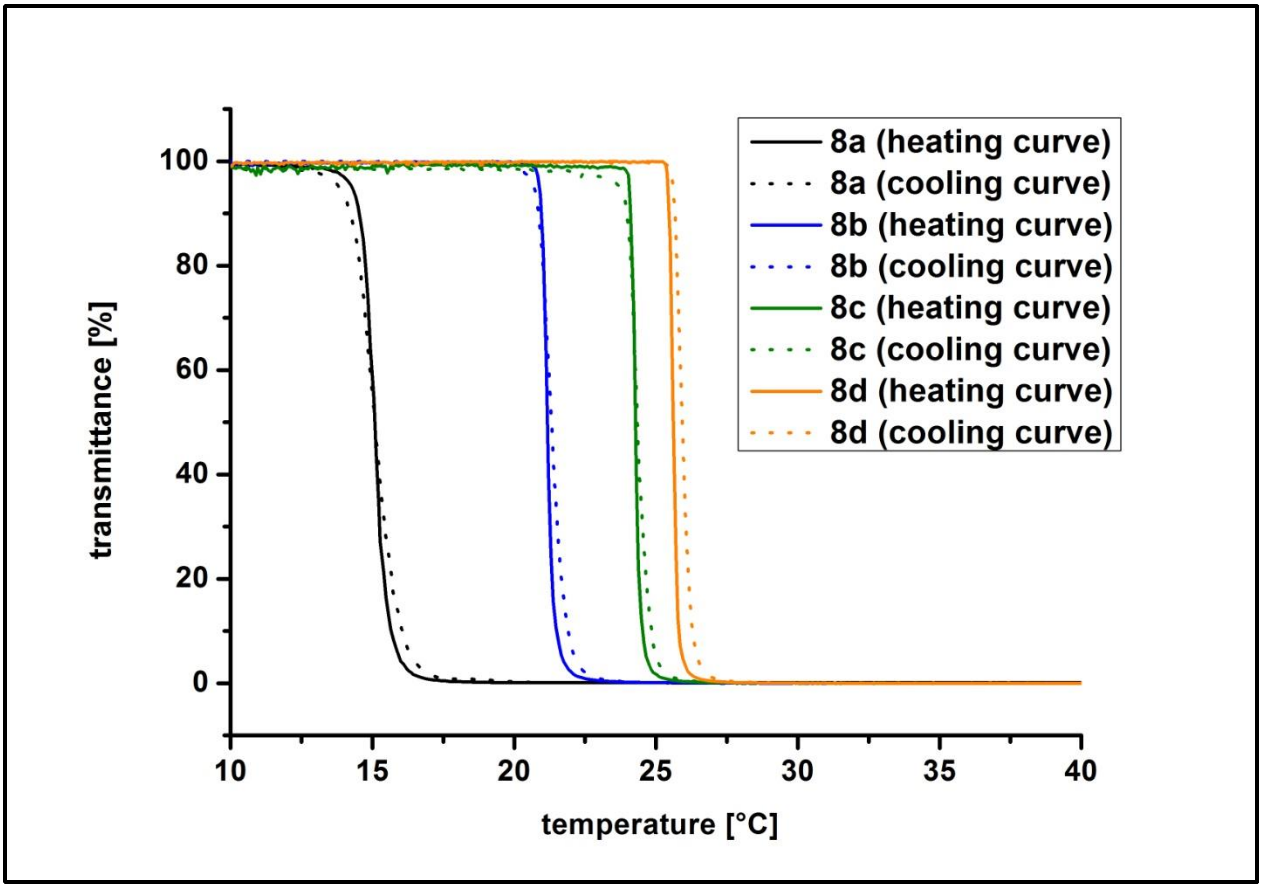

Figure S7: Turbidimetry measurements of *tert*-butyl phenol end-group-functionalized polymers **8a–d** in aqueous solution at a concentration of 10 mg/mL.

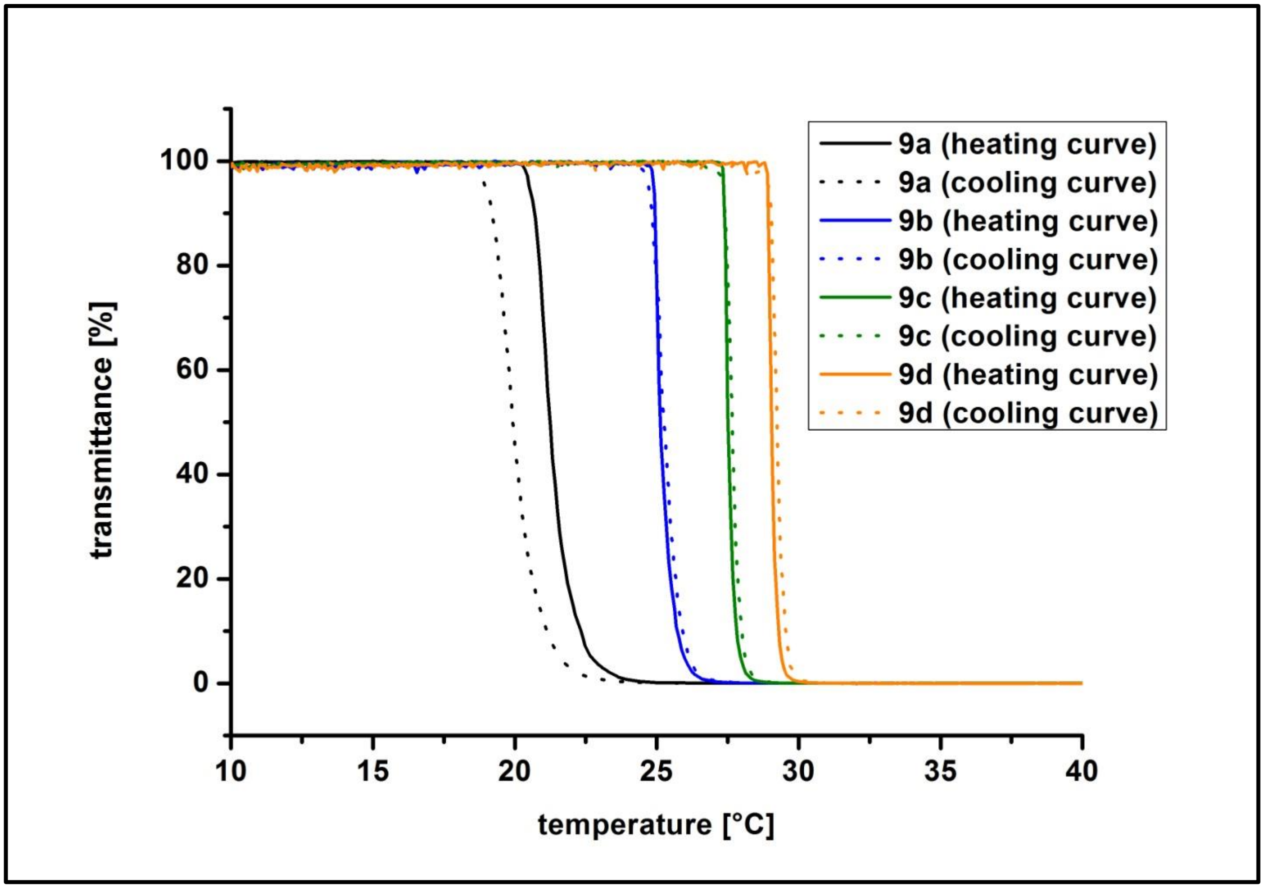

Figure S8: Turbidimetry measurements of *tert*-octyl phenol end-group-functionalized polymers **9a–d** in aqueous solution at a concentration of 10 mg/mL.

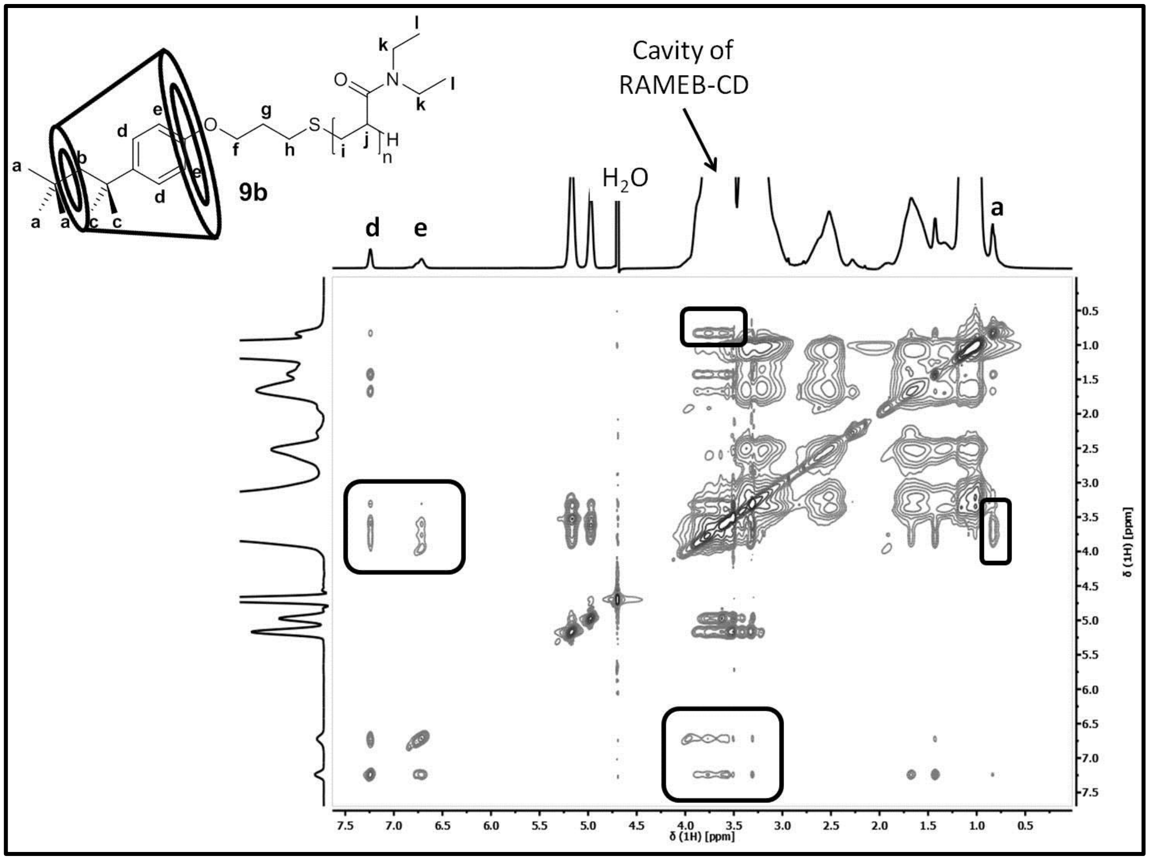

Figure S9: 2D NMR-NOESY spectrum of polymer **9b** with three equivalents RAMEB-CD in D_2O (600 MHz, rt).

References

1. Tsai, T.-W.; Wang, E.-C.; Li, S.-R.; Chen, Y.-H.; Lin, Y.-L.; Wang, Y.-F.; Huang, K.-S. *J. Chin. Chem. Soc.* **2004**, *51*, 1307-1318.
2. Kornblum, N.; Lurie, A. P. *J. Am. Chem. Soc.* **1959**, *81*, 2705-2715.
3. Mayo, F. R. *J. Am. Chem. Soc.* **1943**, *65*, 2324-2329.
4. Matyjaszewski, K.; Davis, T. P. Handbook of radical polymerization; Wiley Online Library, 2002.
